# Immunization with the Gly^1127^-Cys^1140^ amino acid sequence of the LRP1 receptor reduces atherosclerosis in rabbits. Molecular, immunohistochemical and nuclear imaging studies

**DOI:** 10.7150/thno.37305

**Published:** 2020-02-10

**Authors:** Olga Bornachea, Aleyda Benitez-Amaro, Angela Vea, Laura Nasarre, David de Gonzalo-Calvo, Juan Carlos Escola-Gil, Lidia Cedo, Antoni Iborra, Laura Martínez-Martínez, Candido Juarez, Juan Antonio Camara, Carina Espinet, Maria Borrell-Pages, Lina Badimon, Joan Castell, Vicenta Llorente-Cortés

**Affiliations:** 1 Institute of Biomedical Research of Barcelona (IIBB). Spanish National Research Council (CSIC), Barcelona, Spain.; 2 Lipids and Cardiovascular Pathology. Biomedical Research Institute Sant Pau (IIB Sant Pau), Hospital de la Santa Creu i Sant Pau. Barcelona. Spain.; 3 CIBER enfermedades cardiovasculares (CIBERcv).; 4 Metabolic Basis of Cardiovascular Risk, Biomedical Research Institute Sant Pau (IIB Sant Pau), Hospital de la Santa Creu i Sant Pau. CIBER de Diabetes y enfermedades Metabólicas Asociadas (CIBERDEM), Barcelona. Spain.; 5 SCAC, Universitat Autònoma de Barcelona (UAB), Bellaterra, Spain; 6 Department of Immunology, Institut de Recerca and Hospital Santa Creu i Sant Pau, Barcelona, Spain.; 7 Preclinical Imaging Platform. Vall dHebron Institute of Research. Barcelona, Spain.; 8 Department of Nuclear Medicine, Institut de Diagnòstic per la Imatge (IDI), Hospital General Universitari Vall d'Hebrón, Universitat Autònoma de Barcelona, Barcelona, Spain.; 9 Cardiovascular Program ICCC, Institut de Recerca Hospital de la Santa Creu i Sant Pau, Barcelona, Spain; 10 Cardiovascular Research Chair, UAB, Barcelona, Spain

**Keywords:** LRP1 (CR9), atherosclerosis, LDL, vascular cholesteryl esters, NF-kB, TNFR1, ^18^F-FDG-PET/CT, Doppler ultrasonography, inflammation

## Abstract

**Background**: The LRP1 (CR9) domain and, in particular, the sequence Gly^1127^-Cys^1140^ (P3) plays a critical role in the binding and internalization of aggregated LDL (agLDL). We aimed to evaluate whether immunization with P3 reduces high-fat diet (HFD)-induced atherosclerosis.

**Methods**: Female New Zealand White (NZW) rabbits were immunized with a primary injection and four reminder doses (R1-R4) of IrP (irrelevant peptide) or P3 conjugated to the carrier. IrP and P3-immunized rabbits were randomly divided into a normal diet group and a HFD-fed group. Anti-P3 antibody levels were determined by ELISA. Lipoprotein profile, circulating and tissue lipids, and vascular pro-inflammatory mediators were determined using standardized methods while atherosclerosis was determined by confocal microscopy studies and non-invasive imaging (PET/CT and Doppler ultrasonography). Studies treating human macrophages (hMΦ) and coronary vascular smooth muscle cells (hcVSMC) with rabbit serums were performed to ascertain the potential impact of anti-P3 Abs on the functionality of these crucial cells.

**Results**: P3 immunization specifically induced the production of anti-P3 antibodies (Abs) and did not alter the lipoprotein profile. HFD strongly induced cholesteryl ester (CE) accumulation in the aorta of both the control and IrP groups, and their serum dose-dependently raised the intracellular CE of hMΦ and hcVSMC, promoting TNFR1 and phospho-NF-kB (p65) overexpression. These HFD pro-inflammatory effects were dramatically decreased in the aorta of P3-immunized rabbits and in hMΦ and hcVSMC exposed to the P3 rabbit serums. Microscopy studies revealed that P3 immunization reduced the percentage of lipids, macrophages, and SMCs in the arterial intima, as well as the atherosclerotic extent and lesion area in the aorta. PET/CT and Doppler ultrasonography studies showed that the average standardized uptake value (SUV_mean_) of the aorta and the arterial resistance index (ARI) of the carotids were more upregulated by HFD in the control and IrP groups than the P3 group.

**Conclusions**: P3 immunization counteracts HFD-induced fatty streak formation in rabbits. The specific blockade of the LRP1 (CR9) domain with Anti-P3 Abs dramatically reduces HFD-induced intracellular CE loading and harmful coupling to pro-inflammatory signaling in the vasculature.

## Introduction

Cardiovascular disease (CVD) is the leading cause of death, the dominant cause of which is atherosclerosis, which mainly occurs in the intima of several middle-sized and large arteries. Intimal thickening, occurring during the first step of atherosclerosis, is majorly associated with low density lipoprotein (LDL) retention by the proteoglycans of the arterial intima. Intimal retained LDLs undergo LDL aggregation (agLDL) and fusion which is a key event in the progression of atherosclerosis. 1-3. AgLDL interacts with the receptor low-density lipoprotein receptor-related protein 1 (LRP1), and LRP1-mediated agLDL uptake causes intracellular cholesteryl ester (CE) loading and foam cell formation in human coronary vascular smooth muscle cells 4-6 and macrophages 7,8.

LRP1 receptor is a signaling mediator that exerts essential cytoprotective and anti-inflammatory functions. Therefore, its potential pathological functions could not be overcome by LRP1 silencing. This strategy will indeed cause deleterious effects in macrophage and VSMC functionality, which both depend on proper LRP1 β-chain signaling 9-11. LRP1 β-chain is phosphorylated on the distal NPxY motif of the by PDGF-BB mitogenic signaling, and this modulates VSMC proliferation^12^,^13^. LRP1 signaling is also essential to limit lesional apoptosis and inflammation through the regulation of monocyte recruitment, macrophage apoptosis susceptibility, and efferocytosis 14,15. In line, it has been thoroughly shown that disabling LRP1 tyrosine phosphorylation causes macrophage intracellular lipid accumulation and the anomalous clearance of apoptotic cells, resulting in accelerated atherosclerosis 16. In addition to signaling mediated by the LRP1 b chain, signaling induced through the binding of its a-chain to extracellular ligands also seems to be essential for optimal vascular function. The atheroprotective induction of Wnt5a mediated by extracellular TGF-β in macrophages requires LRP1 α chain 17. Apolipoprotein E-mediated interleukin-1 receptor associated kinase-1 (IRAK-1) signaling that downregulates NF-κB-induced inflammation requires the binding of Apolipoprotein E to LRP1 α chain 18. The binding of protease-inhibitor complexes to LRP1 α chain inhibit the NF-kB inflammatory response, and induce a cytoprotective effect through phosphorylation of Akt and ERK1/2 protein kinases 19-21. NF-kB is a key player involved in the development and progression of inflammation in different tissues, including the vasculature 22. A vascular mediator in NF-kB activation is tumor necrosis factor-alpha receptor -1 (TNFR1), which enhances arterial wall chemokine and adhesion molecule expression, as well as smooth muscle cell proliferation and migration in atherosclerosis 23.

We have previously identified one peptide sequence (P3: H-GDNDSEDNSDEENC-NH2) (Gly1127-Cys1140) in the CR9 domain of LRP1 that is crucial for its interaction with aggregated LDL 24. We showed that polyclonal antibodies generated against P3 sequence (Anti-P3 Abs), located in the CR9 domain, efficiently blocked hcVSMC-foam cell formation. It is important to note that the CR8/CR9 tandem is the only pair of consecutive CR modules from the LRP1 cluster II that shows only negligible affinity for serpins such as plasminogen activator inhibitor-1 (PAI-1) and protease nexin 1 (PN1) 25. This makes CR9 an ideal target to counteract LRP1-AgLDL interactions without altering the binding of other essential ligands, such as protease-inhibitor complexes, which are key inhibitors of pro-inflammatory signals.

On the basis of these previous results, we considered a challenge within the cardiovascular field to study the anti-atherosclerotic efficacy of anti-P3 antibodies in a translational *in vivo* model, similar to humans in the cholesterol-carried lipoprotein profile. In the rabbit model of HFD-induced atherosclerosis, cholesteryl esters are mainly carried by ApoB-100 lipoproteins and there is an elevated participation of SMCs in fatty streak lesions 26. In addition, this model has been previously validated to study the effects of HDL on fatty streak formation and evolution 27 as well as to study vascular inflammation by the mainstay imaging technique ^18^F-FDG/PET 28.

The aim of this work was to study the potential therapeutic relevance of a LRP1 (CR9)-specific blockade with anti-P3 Ab to counteract HFD-induced atherosclerosis. Our results showed that anti-P3 antibodies reduced HFD-induced cholesteryl ester accumulation and pro-inflammatory signaling in the aorta. The potent anti-inflammatory efficacy of anti-P3 Abs allowed for the corroboration of the treatment's efficacy via non-invasive imaging techniques, such as ^18^F-FDG/PET and Doppler ultrasonography, which provided a high translational impulse to this innovative, anti-atherosclerotic, potentially therapeutic tool.

## Methods

### Peptide Synthesis and conjugation

The P3 peptide used to immunize rabbits contained the following sequence GDNDSEDNSDEENC corresponding to the amino acids 1127 to 1140 located in LRP1 cluster II (domain CR9) 24. The P3 sequence corresponds to an area of high homology between human and rabbit LRP1, with the difference that the asparagine (N) in humans was replaced by a serine (S) in the rabbit protein. In addition, the amino acid C^1148^ in the rabbit sequence (GDNDCEDNSDEENC) was replaced by S to achieve greater peptide immunogenic effectiveness. The irrelevant peptide (IrP) has the same sequence than P3 but with amino acids in D-enantiomer configuration. Both peptides were synthesized by the Laboratory of Proteomics & Protein Chemistry, Department of Experimental & Health Sciences, Pompeu Fabra University, by the solid-phase method using a Prelude peptide synthesizer (Protein Technologies, Inc.). Peptides were purified by high-performance liquid chromatography (HPLC, Waters 600) using UV detection at 254 nanometers (Waters 2487) and characterized by mass spectrometry (Applied Biosystems 4700 Proteomics Analyser).

Peptides were conjugated to the transporter molecule Keyhole limpet haemocyanin (KLH) for immunizations and with bovine serum albumin (BSA) for ELISAs. The conjugation of peptide to KLH and BSA (Sigma, St. Louis, MO) was performed as previously described 29. Peptide-KLH conjugates were used for rabbit immunization and peptide-BSA conjugates for substrate in the immunoassay ELISA to detect specific anti-P3 Abs in the rabbit serum.

### Animal model

Thirty New Zealand White (NZW) rabbits from the San Bernardo Farm animal centre (Navarra, Spain) weighing 1.8-2 kg (6-7 months-age) were used in this study. Rabbits were housed in a Tecniplast R-Suite cage with a surface area of 4.264 cm2. Housing temperature was maintained at 21°C, relative humidity ranged between 40-60%, and the light period was 12 hours a day. All animals had food and water *ad libitum*. Experimental procedures were approved by the Ethics Committee of Animal Experimentation of the Vall d'Hebrón Institute of Research with registration number 46/17, and performed in accordance with Spanish legislation and also with the European Union directives (2010/63/ EU).

Animals were fed the "chow" R-01 diet from Granja San Bernardo with the following formulation: 17.3% protein, 16.7% fibre and 3% fat; or HFD TD.88137 (42% from fat) from ENVIGO. Animals were acclimated for one week before the first immunization and immunized with a primary injection and four reminder doses (R1-R4) of IrP (irrelevant peptide) (IrP group; N=12) or P3 (P3 group; N=15) conjugated to the carrier every 21 days. An additional group of rabbits was injected with carrier alone (control group; N=3). The four doses of IrP or P3 antigen conjugated with KLH (or carrier) were administered subcutaneously (138 μg/kg, maximum volume 150 μL) every 21 days. For the first immunization, IrP or P3-KLH peptides were emulsified in Freund's Adjuvant Complete, and the rest of the immunizations were done using IrP or P3-KLH conjugated in Freund's Incomplete Adjuvant (both from Sigma Aldrich). During the immunization period, the animals were fed a chow diet.

Starting at the R4 time point, IrP and P3-immunized rabbits were randomly divided into normal diet group and high-fat diet (HFD)-fed group. Twelve rabbits (N=6 Irp-injected and N=6 P3-injected) continued fed on the chow diet, whereas fifteen rabbits (N=6 IrP-injected and N=9 P3-injected ) and one rabbit control group (N=3 injected with carrier alone) received HFD for 30 days (supplemental Figure S1). In pre- and post-diet time points, animals were weighed, serum levels of specific anti-P3 Abs were determined by ELISA, and animals were submitted to PET-CT and carotid Doppler ultrasonography imaging studies. After imaging studies, animals were euthanized and aortas, carotids and liver were dissected and processed for molecular, confocal and immunohistochemical studies.

### Determination of circulating lipids and the lipid content in lipoproteins

Total plasma cholesterol, choline-containing phospholipid, triglyceride and NEFA levels were enzymatically determined using commercial kits adapted to a COBAS 501 autoanalyzer (Roche Diagnostics, Rotkreuz, Switzerland). VLDL, LDL and HDL lipoproteins were isolated by sequential ultracentrifugation at 100,000 g for 24 h at a density of 1.006, 1.019-1.063 and 1.063-1.21 g/ml, respectively, using an analytical fixed angle rotor (50.3, Beckman Coulter) 30. The composition of each lipoprotein, including total and free cholesterol, triglycerides and phospholipids, was determined by commercial methods adapted to the COBAS 501 autoanalyzer. Lipoprotein protein concentrations were determined by the bicinchoninic acid method (Termo Scientific, Rockford, IL). Lipoprotein composition was used to calculate the total mass of each lipoprotein.

### Detection of specific antibodies

We standardized immunoELISAs to detect specific antibodies against P3 peptides. Briefly, all sera were analysed using 96-well polystyrene plates (Ref 442404 Maxisorp, NUNC, Labclinics, Spain) coated with peptide-BSA and BSA as a control for the detection of unspecific antibodies. ELISA plates were incubated with several serum dilutions for 90 minutes, and after washes, anti-rabbit IgGs conjugated to peroxidase (Ref 170-6515, BioRad, Spain) was added to detect the antigen-antibody complexes. ELISA was revealed using OPD substrate (Ref P9187, Sigma Aldrich, Spain), and the absorbance was read in a Multilabel reader Victor3 (Perkin Elmer, Turku, Finland) at 450 nm. The absorbances obtained were adjusted to a 4PL curve to calculate the IC50. The parameter 1/IC50 was taken as the antibody titer.

### Isolation of Rabbit Aortic Smooth Muscle Cells

Rabbit aortic smooth muscle cells** (**rSMCs) were obtained by gentle scraping of the medial layer of New Zealand White rabbit aortas after endothelial layer removal. Cells were incubated at 37°C in a humidified atmosphere of 5% CO^2^ in Ham's F12-DMEM (8:2) supplemented with 20% foetal calf serum, 100 U/mL penicillin and 0.1 mg/mL streptomycin. To maintain exponential growth, cells were subcultured by trypsinization and seeded at a density of 10 000 cells/cm^2^. rSMCs were identified by their growth behaviour, morphology, and immunofluorescence. Immunocytochemical identification of cells was performed by specific monoclonal antibodies for α-smooth muscle cell and von Willebrand factor. Cells were fixed with ice-cold methanol at 20°C for 5 minutes. A solution of BSA at 1% in PBS was used as a blocking agent. Monoclonal antibodies against LRP1 (Fitzgerald, 10R-L107c) and polyclonal antibodies obtained from P3-immunized rabbits were diluted in PBS/1% BSA/0.01% Triton X-100. Finally, FITC-conjugated goat anti-mouse IgG and FITC-conjugated goat anti-rabbit IgG were used as secondary antibodies. Images were captured and analysed on a Leica inverted fluorescence confocal microscope (Leica TCS SP2-AOBS).

### Isolation of human macrophages primary cultures

Human macrophages (hMΦ) were obtained from buffy coats of healthy blood donors. Cells were applied on 15 ml of Ficoll-Hypaque and centrifuged at 300 g for 1 hour at 22°C, with no brake. Mononuclear cells were obtained from the central white band of the gradient, exhaustively washed in Dulbecco's phosphate buffer saline, and suspended in RPMI medium (Gibco) supplemented with 10% human serum AB (Sigma). Cells were left 7 days in culture and allowed to differentiate into macrophages by changing the cell culture media (RPMI supplemented with 10% human serum AB, 100 units/ml penicillin and 100 µg/ml streptomycin) every 3 days.

### Culture of coronary human vascular smooth muscle cells

Human coronary vascular smooth muscle cells (hVSMC) were obtained from the medium layer isolated from macroscopically healthy coronary artery segments collected from patients undergoing cardiac transplantation at Hospital de la Santa Creu i Sant Pau (Barcelona, Spain). hVSMCs were isolated by a modification of the explant technique, as described previously 4-6. The explants were incubated at 37 °C in a humidified atmosphere of 5% CO2. After 1week, the cells start to migrate from the explants and proliferate, covering the floor of the culture well. The medium was exchanged every 3 days after the onset of cell outgrowth; a significant outgrowth was reached after 10 days. Tissue fragments were collected with forceps and placed in a new dish with fresh medium. The cells that remained in the dish were cultured until confluence. For characterization, cells were seeded in cover-slips and grown to confluence. Cell quiescence was induced by maintaining the cell culture for 24 h in a medium with 0.2% FCS or for 48 h in a medium with 0.4% serum. All experiments used serum-deprived cells between passages 4 and 6; VSMCs at these passages appeared as a relatively homogeneous population, showing a hill-and-valley pattern at confluence. Western blot analysis for specific differentiation markers revealed high levels of-actin (45 kDa) and calponin (33 kDa). Cell monolayers were grown in medium 199 supplemented with 20% FBS, 2% human serum, 2 mmol/literL-glutamine, 100 units/ml penicillin G, and 100g/ml streptomycin. The study was approved by the Institutional Ethics Committee at Hospital de la Santa Creu i Sant Pau and conducted in accordance with the Declaration of Helsinki.

### Treatment of hMΦ and hcVSMC with rabbit serums

Quiescent hMΦ and hcVSMC were exposed to increasing concentrations of serum (0.25%, 0.5% and 1%) from the different rabbit groups for two hours. In some experiments, quiescent hMΦ were exposed to aggregated LDL (100 µg/mL, 2 hours), generated as previously described 4-6 in the presence of increasing concentrations (1%, 5% and 10%) of chow serum from the different group of rabbits. Following the serum incubation period, cells were exhaustively washed and harvested in NaOH 0.1 M or lysis buffer for lipidic and Western blot analysis.

### ^18^F-FDG PET/CT and image analysis

An injection of ^18^F-FDG (0.5 mCi/kg) was administered through a catheter in the marginal vein of the ear of each animal. The image study was performed on a Siemens Biograph mCT S64 hybrid PET/CT instrument. We carried out an angiographic PET/CT study at 120 and 180 minutes, which allowed the anatomical and metabolic localization of the studied structures. The CT and PET studies were recorded for 5-6 seconds and 15 minutes, respectively.

#### Acquisition parameters

Thoracic-abdominal imaging studies of the heart, great vessels and abdominal aorta in only one bed position (15 minutes) were performed at the maximum resolution of the PET/CT equipment (1.5 mm in the PET and 0.5 mm in the CT).

#### CT characteristics (attenuation correction)

AngioCT characteristics: An angiographic study was performed before PET acquisition. VISIPAQUE^TM^ (iodixanol) contrast was used and injected at 0.7ml/sec.

#### Image analyses

The co-registration of both studies (PET and CT) was automatically carried out by the Software of the Siemens Biograph mCT S64 hybrid PET/CT equipment. PET/CT data were displayed in axial planes on a Syngovia (Siemens) workstation. Mean standard uptake value (SUV)s were recorded on contiguous axial slices of the aorta superior, central and inferior (one ROI in each area).

The corresponding SUV units were calculated in each animal. These units allow the comparison and standardization of the emission values of each structure studied, including decay correction between dual time-point images. PET/CT outcome variables were the maximum and averaged SUVs.

### Ultrasonography

Ultrasonography was performed with a Mindray M7 device (Shenzen Mindray Bio-medical Electronics, Shenzen, PRC) and a 7.5 MHz central frequency lineal probe. All animals were anaesthetized with propofol and maintained with iIsofluorane (2-3% in fresh air) to respect their welfare and to avoid tachycardia or stress-induced pulse frequency.

Ultrasonographic exams were performed for carotid arteries. The morphology of carotid arteries was measured using B mode images, followed by colour and pulsed wave Doppler analysis to check the blood flow. The specific location at the internal and external carotid division was selected following previously published reference 31, assuming that this location is a target for lipid deposits.

The parameters included were systolic and diastolic velocities, as well as arterial resistance index (ARI). ARI=(Systolic velocity-Diastolic velocity)/Systolic velocity). In all the ultrasonographic parameters, three different measurements were performed in each image, and the mean value was used for parameter calculation. The software used for measurements was that included in the ultrasound device, and mathematical calculations were obtained in Excel format.

### Preparation of histological sections and immunohistochemistry

At necropsy, the aorta, carotids and liver were dissected. The aorta was cleaned from external fat and divided into two sections: thoracic aorta, from the aortic arc to the hepatic aorta, and abdominal aorta, from the hepatic aorta to the bifurcation of the iliac aortas (Supplemental Figure S2). As shown in Supplemental Figure 2, the first 2 cm section of the thoracic aorta was opened by a longitudinal incision. The rest of the aorta was divided into 1cm pieces. Alternatively, each piece was frozen and sectioned for histological and molecular studies. Carotids were frozen and kept at -80 ºC for further analysis of lipid composition.

Longitudinally open sections of aorta were fixed in 4% paraformaldehyde-PBS solution for fatty streak detection and quantification using Herxheimer's staining method. Samples for immunohistochemistry were transversally cut with a cryostat in serial sections of 5 μm and mounted in gelatinized slides. Transversally cut sections were also stained with Herxheimer's staining method to detect lipids. Images were captured with an Olympus Vanox AHBT3 microscope, and digitalized using a Sony 3CCD camera.

### Confocal microscopy studies

Arteries were dissected, immersed in cell maintenance media, examined under low magnification with a zoom stereo microscope and classified according to the criteria of the American Heart Association (AHA). At this point, samples were used for immunohistochemistry or were stained with Masson's trichromic to identify vascular structures. Serial sections (5 μm thick) were immunostained with Phalloidin-488 (to identify smooth muscle cells), and RAM11 (Dako M063301-8), NF-kB p65 (C22B4) (Cell Signalling; #4764) and TNFR1 (MyBiosource MBS840939) with secondary antibody Alexa Fluor 633 IgG). Images were recorded on a Leica inverted fluorescence confocal microscope (Leica TCS SP2-AOBS, Germany). Arteries were viewed with HCX PL APO 20x/0,75 IMM Corr CS2 objective. Fluorescent images were acquired in a scan format of 1024 x 1024 pixels and were processed with the Leica Standard Software TCS-AOBS. Controls without primary antibody showed no fluorescence labelling.

### Image analysis

Image analysis was performed using Visilog 5.4 software (Noesis, France) to quantify the extent of the lesions, and the results were given as the percentage of the total aortic area covered by lipid. Images were captured with a Vanox AHBT3 microscope (Olympus) and digitized using a DXC-S500 camera (Sony) at x200 magnification. Six different sections were analysed per animal, and six animals were analysed per group. The results were shown as the mean ± SD.

### Western blotting analysis

Blots were incubated with antibodies against mouse LRP1 (β*-*chain, clone 5A6 RDI-PRO61066), SREBP-2 (Santa Cruz Biotechnology; sc-13552), phospho-NF-kB p65 (Cell Signaling; #3039), NF-kB p65 (C22B4) (Cell Signalling; #4764) and TNFR1 (MyBiosource MBS840939) Protein extracts (10 μg) were loaded, resolved on 12% SDS-PAGE and transferred to nitrocellulose membranes (BioRad). Signal detection was carried out with the ECL immunoblotting detection system (GE Healthcare) and the results were quantitatively analyzed using Chemidoc (BioRad). Equal protein loading in each lane was verified by incubating blots with monoclonal antibodies against β-actine (Cell Signaling Technology, Inc, #3700S).

Lipid analyses and determination of aortic, carotid and hepatic neutral lipid content Aorta (10 mg), liver (2.5 mg) and the whole extracted carotid were homogenized in NaOH 0.1 M. Lipids were extracted and quantified as previously described 4-6. Cholesteryl esters (CE) triglycerides (TG) and free cholesterol (FC) were analysed by thin-layer chromatography (TLC). The organic solvent was removed under an N_2_ stream, the lipid extract was redissolved in dichloromethane, and one aliquot was partitioned by TLC which was performed on silica G-24 plates. Different concentrations of standard (a mixture of cholesterol, triglycerides and cholesterol palmitate) were applied to each plate. The chromatographic developing solution was heptane/diethylether/acetic acid (74:21:4, vol/vol/vol). Spots corresponding to CE and FC were quantified by densitometry against the standard curve of cholesterol palmitate and cholesterol, respectively, using a computing densitometer.

### Statistical analysis

The results were expressed as the mean ± SD. Differences between study groups were analysed using one-way analysis of variance (ANOVA) followed by a *post-hoc Tukey b-*test. In the image analysis, Student's *t*-test for paired samples was used to compare differences between baseline and HFD feeding PET/CT parameters in each experimental group. The statistical software R (www.r-project.org) was used for all statistical analyses. Differences were considered statistically significant when *P* < 0.05.

## Results

### P3- Immunization induces the production of anti-P3 antibodies in rabbits

**Figure 1:**
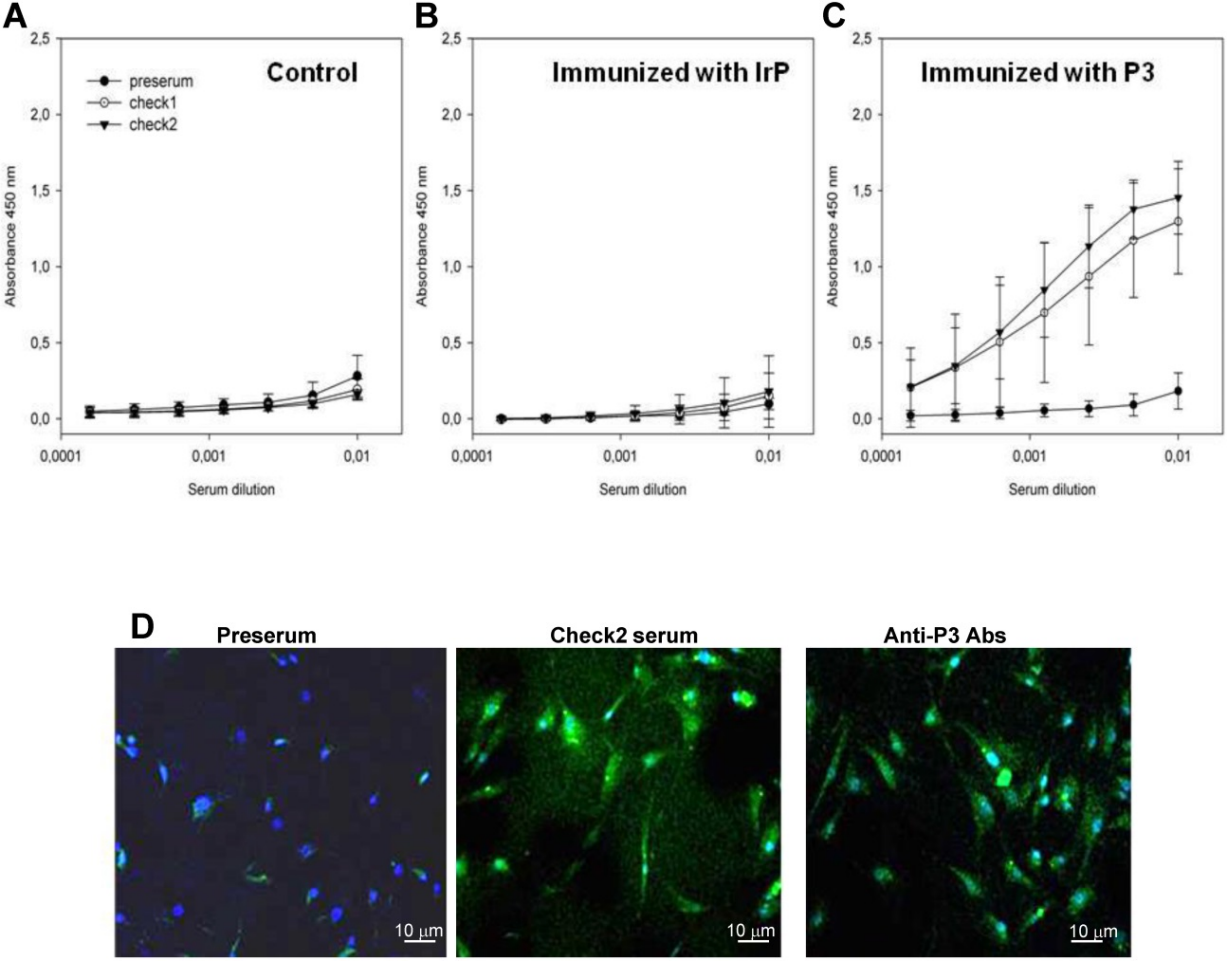
** P3 immunization raises serum levels of specific anti-P3 antibodies.** (**A**) Graphs showing the results of standardized enzyme-linked immunosorbent assay (ELISA) in pre-serum and serum at the check points (Check1 and Check2) from control (**A**), IrP- (**B**) and P3-treated rabbits (**C**) against P3-BSA used as antigenic source. Results are shown as mean±SD. Control (N=3); IrP (N=12); P3 (N=15) (**D**) Confocal images of rabbit SMCs exposed to serum from P3-treated rabbits (middle panel) or to commercial anti-LRP1 (right panel).

ELISA analyses showed the absence of specific antibodies against P3 in the serum of both the control (Figure 1A) and IrP-injected groups (Figure 1B) as well as its presence in P3-immunized rabbits serum (Figure 1C). Anti-P3 Abs levels were maintained in P3-immunized rabbits serum throughout the entire diet period. Previous studies focusing on the functional evaluation of anti-P3 Abs showed their efficacy in reducing foam cell formation through the blockade of the LRP1/agLDL interaction 24. Here, confocal microscopy studies revealed that Abs in the P3-immunized rabbits serum hybridized with the LRP1 with a similar efficiency as commercial anti-LRP1 Abs in rabbit aortic vascular smooth muscle cells (rSMCs) (Figure 1D). None of the sera showed an unspecific response when only BSA without a conjugated peptide, was used as an antigenic source in ELISA (data not shown).

### Anti-P3 Abs counteract HFD-induced atherosclerosis in rabbits

**Figure 2:**
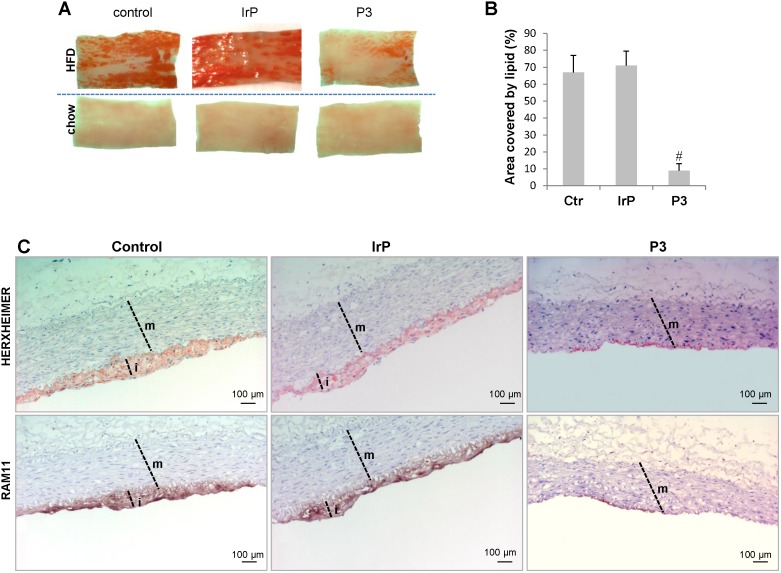
** P3 immunization reduces HFD-induced atherosclerotic burden.** (**A**) Representative images of longitudinally open sections of aorta stained with Herxheimer in control, IrP and P3-immunized rabbits fed either chow or HFD. (**B**) Graph showing quantification of atherosclerotic burden in HFD-fed rabbits from control (N=3), IrP (N=6) and P3 (N=9). Results are shown as mean±SD of 8 aortic sections/rabbit. #P<0.005, P3 *versus* control and IrP groups. (**C**) Representative immunohistochemical images of lipid and macrophage staining in cross-sections of aortas from control and P3-immunized rabbits, both on HFD m: media; i: intima.

**Figure 3:**
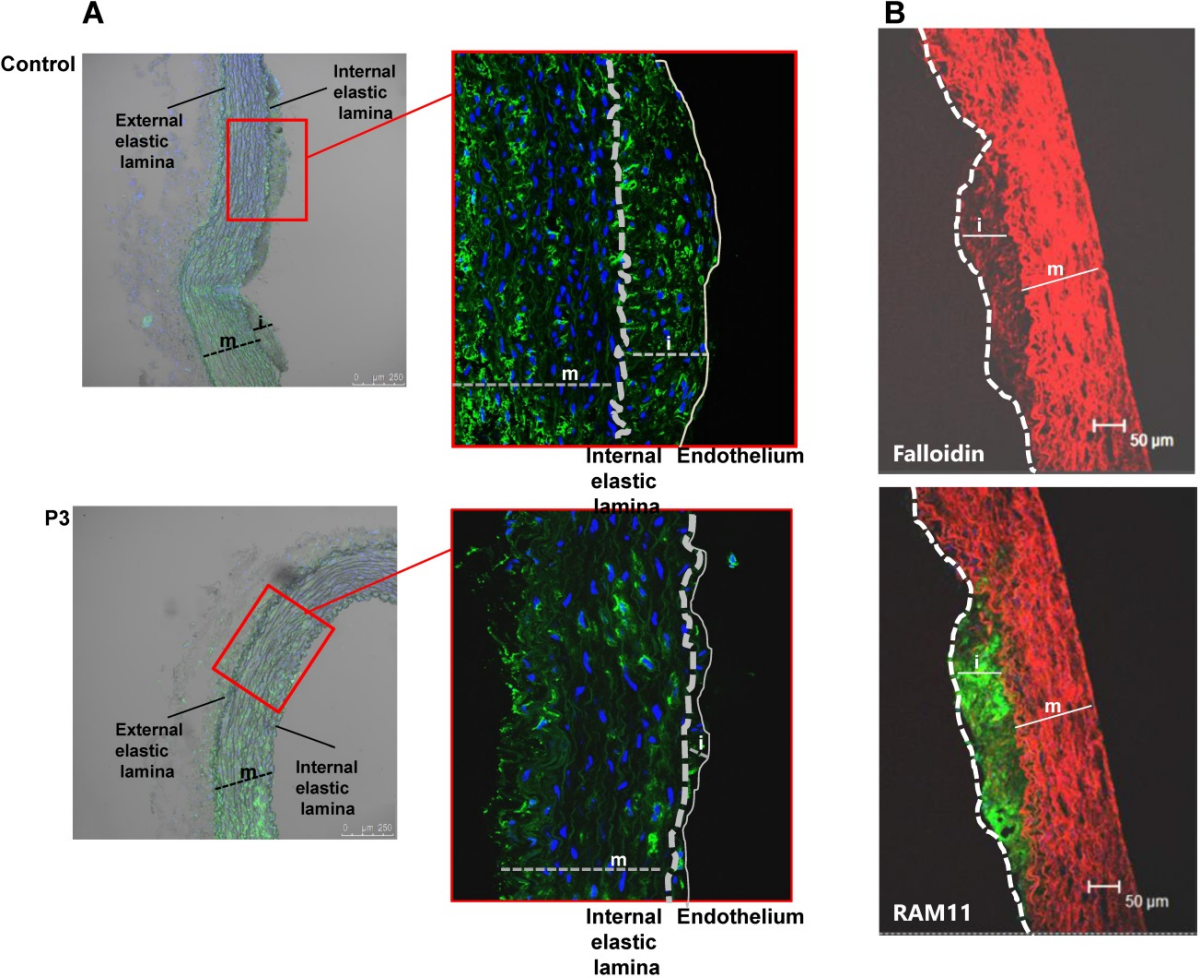
** P3 immunization reduces HFD-induced SMC accumulation in the arterial intima**. Representative confocal microscopy images of SMCs detected with anti-a-actin antibodies in cross-sections of aortas from control and P3- immunized rabbits (**A**) and of SMC detected with falloidin and macrophages detected with anti-RAM antibodies (**B**). Fluorescent images were acquired in a scan format of 1024 x 1024 pixels and were processed with the Leica Standard Software TCS-AOBS. m: media; i: intima.

*En face* preparations of the thoracic aorta showed that HFD feeding in rabbits induced a high percentage of occupation of the aortic vasculature with fatty streak lesions in the control and IrP groups but not in the P3-immunized group (Figure 2A and 2B). Immunohistochemistry studies revealed a high percentage of lipids and macrophages in the arterial intima of control and IrP-immunized rabbits that was strongly reduced in P3-immunized rabbits (Figure 2C). Finally, confocal microscopy studies showed an elevated presence of α-actin positive cells in the intima of the initial lesions in control and IrP groups that was almost completely absent in the P3 group (Figure 3A). Both VSMC (falloidin positive cells in red) and macrophages (RAM11 positive cells in green) coexist in the intimal thickening of initial atherosclerotic lesions (Figure 3B). Taken together, these results demonstrate that anti-P3 Abs limit monocyte recruitment and VSMC migration, thus preventing early atherosclerotic formation in rabbits.

### Anti-P3 Abs reduce cholesteryl ester accumulation and pro-inflammatory signals induced by HFD in the vasculature of rabbits without altering serum lipid levels or the lipoprotein profile

**Figure 4:**
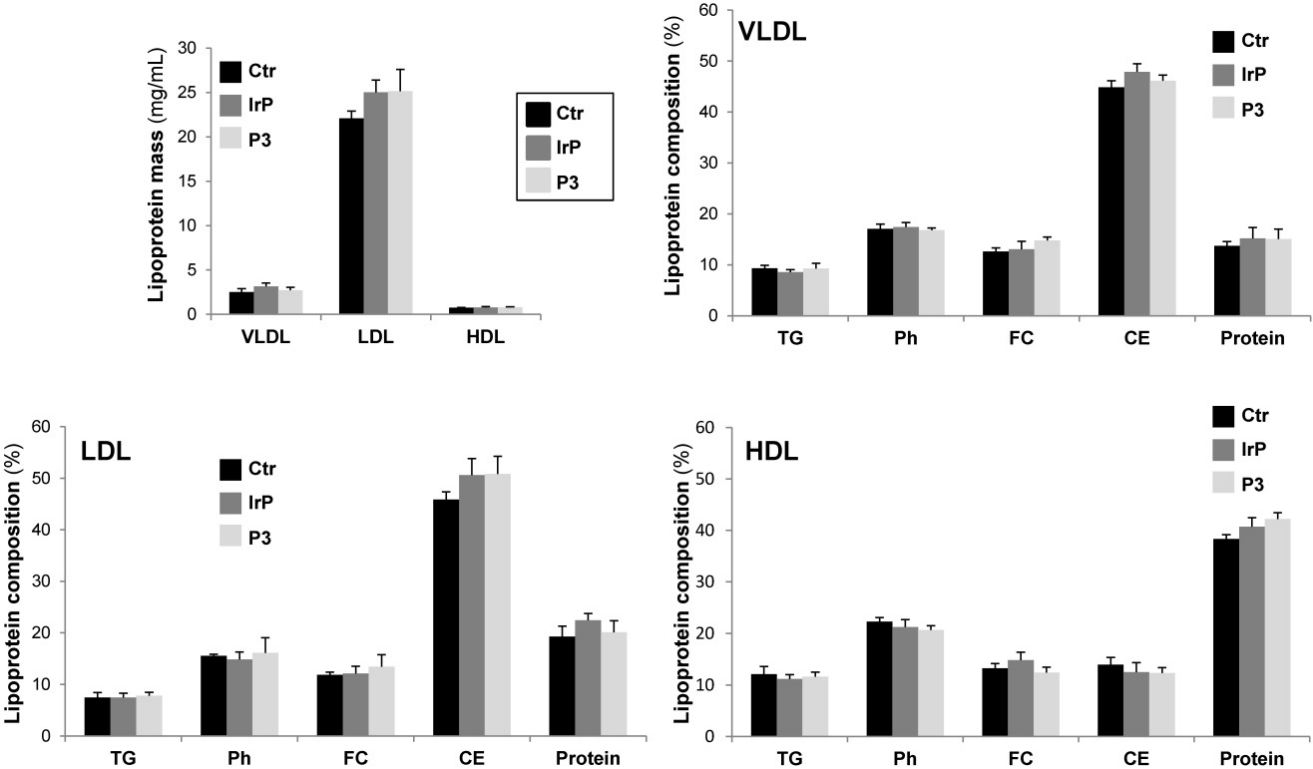
** P3 immunization did not induce alterations in lipoprotein mass subclasses or lipoprotein profile of HFD fed rabbits**. VLDL, LDL and HDL were isolated by sequential ultracentrifugation and their composition in terms of total, free cholesterol, triglycerides, phospholipids and protein were determined as explained in methods. Lipoprotein composition was used to calculate the total mass of each lipoprotein. Results are shown as mean±SD of control (N=3), IrP (N=6) and P3-immunized rabbits (N=9).

Circulating levels of cholesterol, phospholipid and NEFAs, but not triglycerides, were strongly higher in HFD- compared to chow fed rabbits, but there were no differences in P3 compared to control groups (Suplemental Figure S3). VLDL, LDL, or HDL lipoprotein masses and triglyceride (TG), phospholipid (Ph), free cholesterol (FC), cholesteryl esters (CE) and protein levels in lipoprotein fractions were similar among the three groups (Figure 4). These results clearly indicate that P3 immunization did not alter the plasma lipid levels and that, in rabbits, cholesteryl esters are mainly carried by LDL and VLDL lipoproteins in all rabbit groups. There were no differences on weight gain according to diet or between groups (supplemental Figure S4).

**Figure 5:**
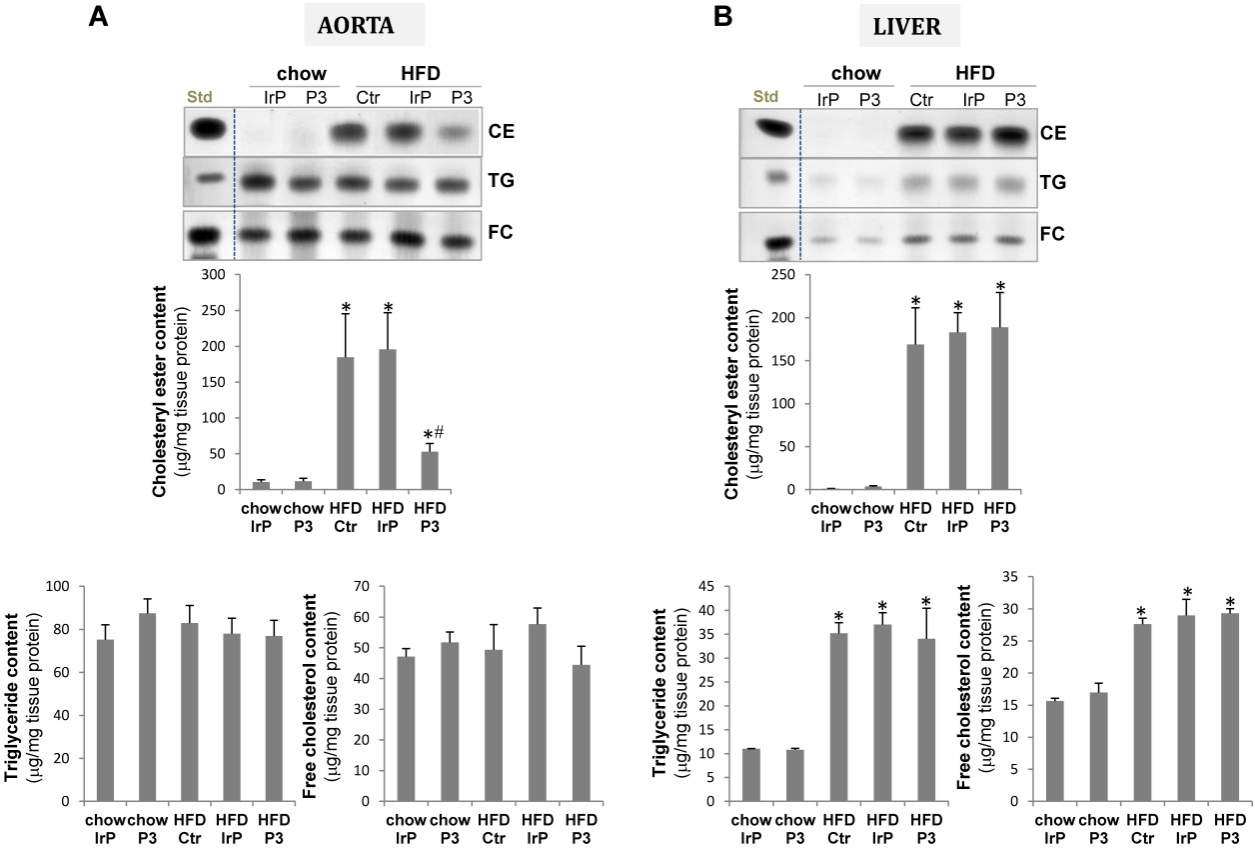
** P3 immunization reduces HFD-induced cholesterol accumulation in the aorta but not in the liver.** Representative thin-layer chromatography of aorta (**A**) and liver (**B**). Bar graphs showing the quantification of cholesteryl ester (CE), triglycerides and free cholesterol content of aorta and liver. Results are expressed as µg cholesterol per mg protein and shown as mean ± SD in chow IrP (N=6), chow P3 (N=6), HFD Ctr (N=3), HFD IrP (N=6) and HFD P3 (N=9). *P<0.005 versus chow diet; #P<0.005 versus control or IrP-rabbits.

Consistent with the high impact of HFD on the VLDL and LDL cholesteryl ester content, HFD increased CE accumulation to a much higher extent in the aorta of control and IrP groups than in the P3-immunized group (Figure 5A). There were no differences in the low TG or FC content in the vasculature between groups. In the liver, HFD strongly increased CE, TG and FC content with no differences between the groups (Figure 5B).

**Figure 6:**
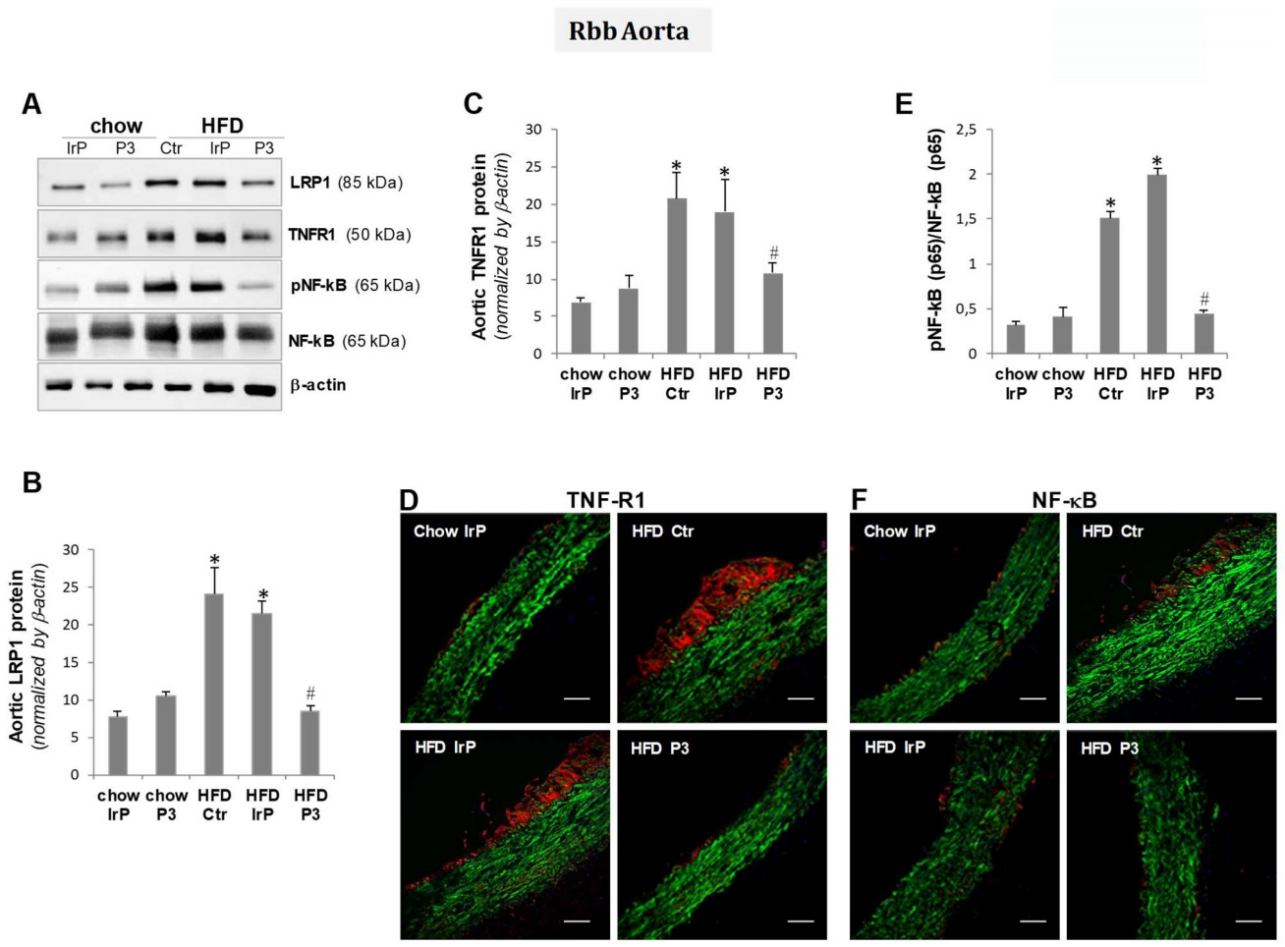
** P3 immunization reduces aortic pro-inflammatory mediators in the aorta**. Representative Western blot analysis of LRP1, TNFR1, pNF-kB (p65), total NF-kB (p65), and β-actin bands (**A**) and bar graphs showing band quantification of LRP1 and TNFR1 normalized by a-actin (**B,C**) and pNF-kB (p65)/total NF-kB (p65) ratio (**E**). Results are shown as mean ± SD in chow IrP (N=6), chow P3 (N=6), HFD Ctr (N=3), HFD IrP (N=6) and HFD P3 (N=9). *P<0.005 versus chow diet; #P<0.005 versus control or IrP-rabbits. Representative confocal microscopy images of TNFR1 (**D**) and NF-kB levels (**F**) in aortic samples from the different groups. Scale bar = 50 µm.

Molecular studies have shown that HFD increased LRP1 levels in the aorta (Figure 6A,B), as previously shown in this and other models by the group 4,32. The upregulatory effect of HFD on LRP1 was not observed in the aorta of P3-immunized rabbits, confirming the vascular LRP1 upregulation by cholesteryl esters in this particular experimental setting.

Molecular studies combined with confocal studies have shown that HFD raises TNFR1 (Figure 6A,C,D)and NF-kB (p65) (Figure 6A,E,F) protein levels in the aorta of the control and IrP groups but not in the P3-immunized group. These results indicate that anti-P3 are highly efficient inhibiting cholesteryl ester accumulation and pro-infammatory signaling in the vasculature of rabbits.

### HFD serum from the P3-immunized rabbits, different from that of the control and IrP-immunized rabbits, failed to induce intracellular cholesteryl ester accumulation and pro-inflammatory signaling in human macrophages and human coronary vascular smooth muscle cells

**Figure 7:**
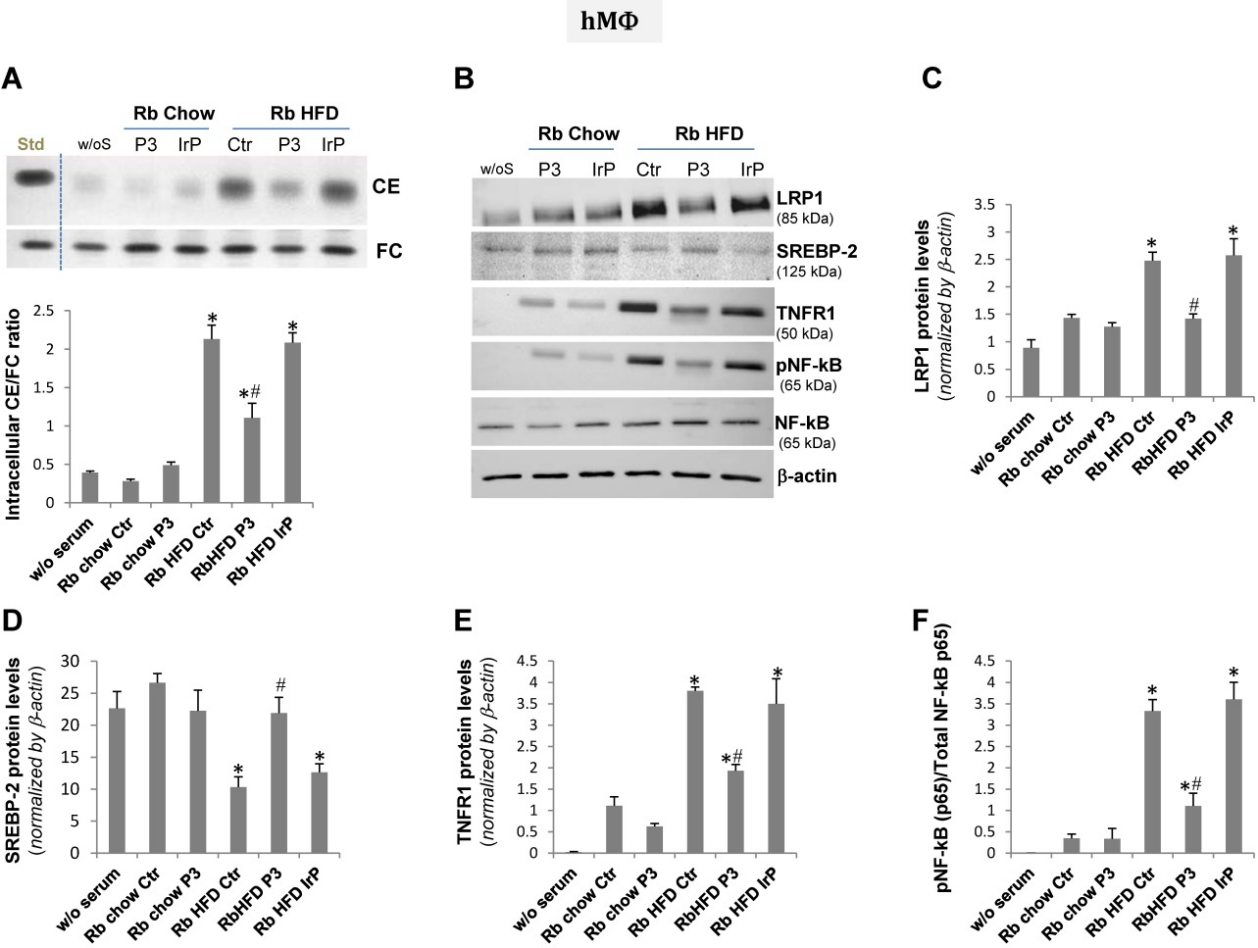
** HFD serum from control and IrP serum, but not P3 serum, dramatically increased intracellular CE and pro-inflammatory mediators in human macrophages**. Quiescent human macrophages (hMΦ) were exposed to serum from the different groups (0.5%, 2 hours). Cells were then exhaustively washed and collected in NaoH 0.1N for lipid extraction or in lysis buffer for Western blot analysis. **A**) Representative TLC and bar graphs showing the cholesteryl ester (CE) /free cholesterol ratio. Representative Western blot analysis of LRP1, SREBP-2, TNFR1, pNF-kB (p65), total NF-kB (p65), and a-actin bands (**B**) and bar graphs showing band quantification of LRP1, SREBP-2 and TNFR1 normalized by a-actin (**C,D,E**) and pNF-kB (p65)/total NF-kB (p65) ratio (**F**). Results are shown as mean ± SD of three experiments performed in duplicate. *P<0.005 versus chow serum; #P<0.005 versus control or IrP-serums.

**Figure 8:**
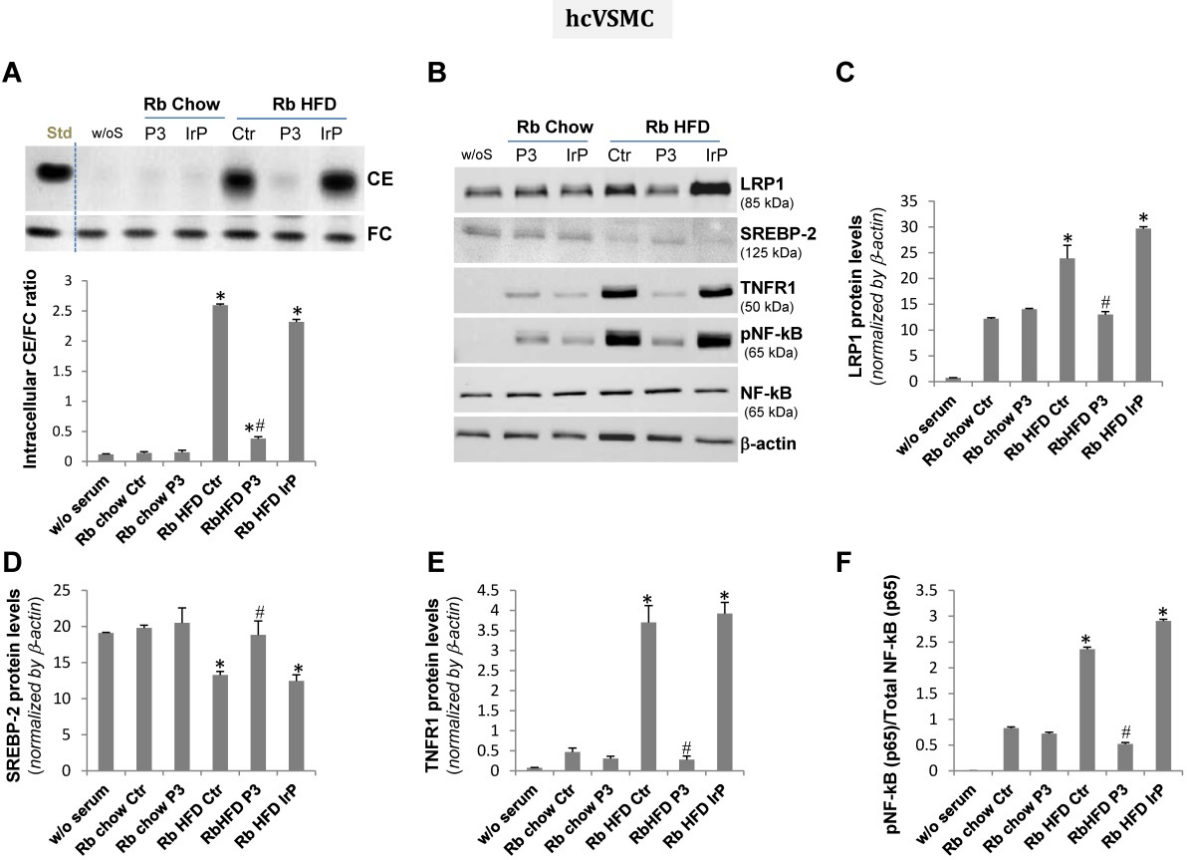
** HFD serum from control and IrP serum, but not P3 serum, dramatically increased intracellular CE and pro-inflammatory mediators in human coronary vascular smooth muscle cells**. Quiescent human coronary vascular smooth muscle cells (hcVSMC) were exposed to serum from the different groups (0.5%, 2 hours). Cells were then exhaustively washed and collected in NaoH 0.1N for lipid extraction or in lysis buffer for Western blot analysis. **A**) Representative TLC and bar graphs showing the cholesteryl ester (CE) /free cholesterol ratio. Representative Western blot analysis of LRP1, SREBP-2, TNFR1, pNF-kB (p65), total NF-kB (p65), and a-actin bands (**B**) and bar graphs showing band quantification of LRP1, SREBP-2 and TNFR1 normalized by a-actin (**C,D,E**) and pNF-kB (p65)/total NF-kB (p65) ratio (**F**). Results are shown as mean ± SD of three experiments performed in duplicate. *P<0.005 versus chow serum; #P<0.005 versus control or IrP-serums.

The treatment of human macrophage** (**hMΦ**)** and human coronary vascular smooth muscle cell (hcVSMC) with chow serum from the control, IrP or P3 groups (1%, 2 hours) failed to increase intracellular cholesteryl ester content in hMΦ (Figure 7A) and hcVSMC (Figure 8A). In contrast, treatment of cells with HFD serum (1%, 2 hours) from the control and IrP groups (but not the P3 group) dramatically raised the intracellular CE content in hMΦ (Figure 7A) and hcVSMC (Figure 8A). HFD serum from control and IrP groups (but not the P3 group) dose-dependently raised the intracellular CE content of both hMΦ and hcVSMC (supplemental Figure S5). Treatment with chow serum from P3-immunized rabbits but not from control or IrP-injected rabbits dose-dependently reduced intracellular CE accumulation in hMΦ exposed to aggregated LDL (100 µg/mL, 2 hours) (supplemental Figure S6).

Concurrently with HFD serum-induced intracellular CE accumulation, LRP1 protein levels were augmented while those of sterol regulatory element binding protein 2 (SREBP-2) decayed in hMΦ and hcVSMC exposed to (control and IrP)-HFD serums (1%, 2 hours) but not in cells exposed to P3 serum under the same experimental conditions (Figure 7B,C,D and 8B,C,D respectively). These results are in line with the capacity of intracellular cholesteryl esters to stimulate LRP1 transcription through SREBP-2 downregulation previously described by our group 4,33,34. There were no differences in LRP1 and SREBP-2 protein levels between cells exposed to chow serum from the different groups.

Our molecular studies showed that HFD serum (from the control and IrP groups) promote a much higher induction of crucial inflammatory mediators, such as TNFR1 and pNF-kB (p65), than a chow diet in hMΦ (Figure 7B,E,F) and hcVSMC (Figure 8B,E,F). However, P3 immunization efficiently restricted the HFD serum pro-inflammatory effects in cells to the levels found in cells exposed to chow serums. These results highlight the crucial role of Anti-P3 Abs in counteracting HFD-induced pro-inflammatory signaling coupled with the intracellular cholesteryl ester loading of human macrophages and human coronary vascular smooth muscle cells.

### PET/CT imaging studies show that Anti-P3 Abs reduce ^18^F-FDG uptake in the aorta of rabbits

**Figure 9:**
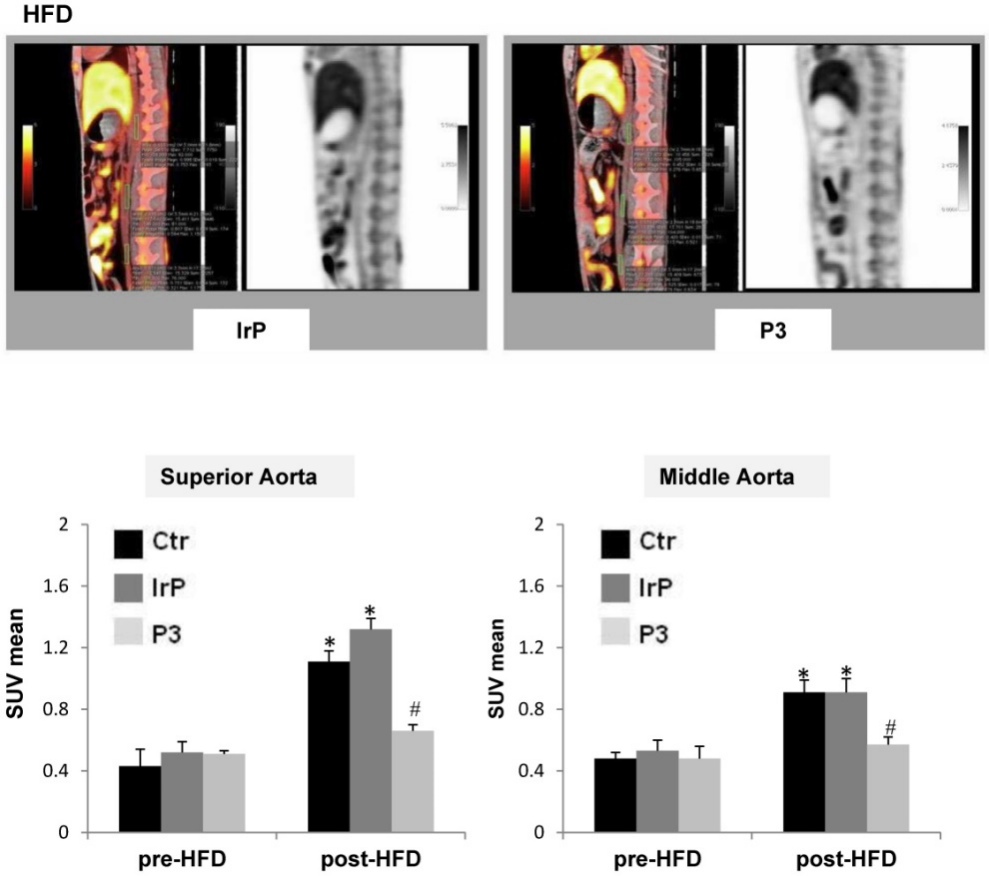
** P3 immunization counteracts aortic ^18^F-FDG uptake induced by HFD in rabbits.** (**A**) Representative PET/CT longitudinal images of the aorta in control and P3 immunized rabbits fed HFD. (**B**) Graphs showing the SUV_mean_ progression from pre-diet to post-diet time points in the upper and middle parts of the aorta in HFD-fed rabbits (IrP and P3 groups) (N=6/group). *P<0.005 versus pred-diet; #P<0.005 versus IrP-rabbits.

PET-CT metabolic images were performed and analyzed at 120 and 180 minutes after the intravenous administration of 18-FDG in the three groups before their submission to a specific diet. After 31 days of specific diet feeding, subsequent new metabolic images were obtained and analyzed at 120 and 180 minutes. Maximal-intensity projections (MIP) and orthogonal slices were analyzed in fused morpho-metabolic images to identify the aortic tract, and, in the axial plane, three sections were selected for SUV measurements: upper, middle and lower. A circular region of interest (ROI) was drawn following anatomical limits in each section, from which SUV_mean_ was obtained. A progressive decrease in aortic activity was observed as a function of time, which was attributed to the clearance of the vascular background activity. The acquisition that best separated the groups was that performed at 120 minutes. The measurements in the three segments of the aorta showed that the highest ^18^F-FDG uptake activity occurred in the upper region while it decreased in the middle region and practically disappeared in the lower region. HFD significantly increased SUV_mean_ in the upper and middle regions of the aorta of control groups, as previously shown 35. In contrast, in the P3-immunized rabbits, HFD only slightly induced SUV_mean_ in these aortic regions (Figure 9). Moreover, rabbits on the chow diet did not show alterations in post-diet *versus* pre-diet SUV_mean_ values (supplemental Figure S7). The high impact of Anti-P3 Abs on aortic ^18^F-FDG uptake confirmed molecular, confocal and immunohistochemical results obtained in the present study showing an elevated anti-inflammatory potential of anti-P3 Abs in macrophages and smooth muscle cells in the vasculature.

### Doppler ultrasonography imaging reveals that anti-P3 Abs prevent the HFD-induced arterial resistance index in the carotids of rabbits

**Figure 10:**
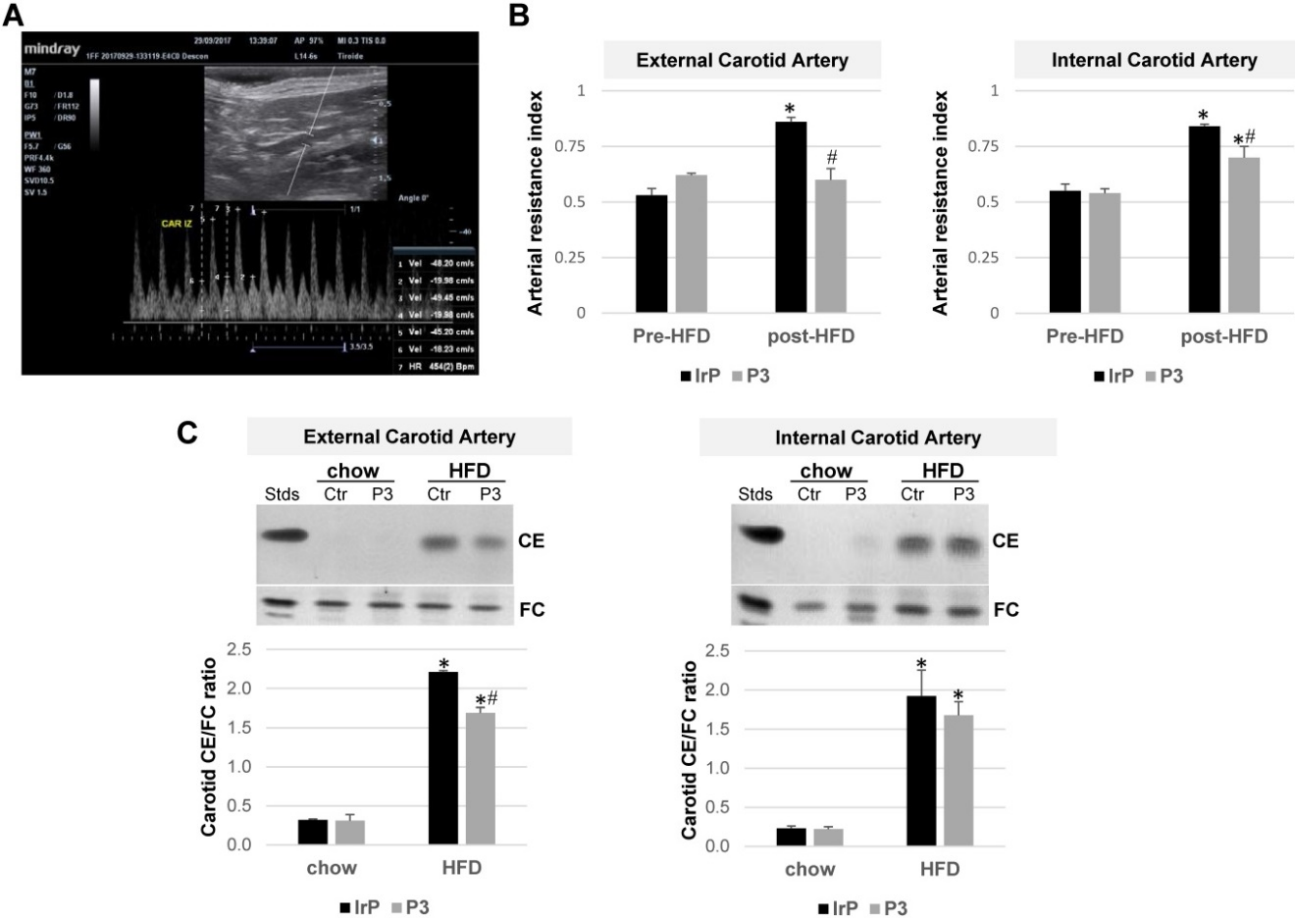
** P3 immunization reduces the HFD-induced arterial resistance index (RI) and cholesteryl ester content in the carotids of rabbits.** (**A**) Representative image of Eco Pulsed-Wave Doppler in the external carotid of rabbits. The systolic (1, 3 and 5), diastolic (2, 4 and 6), and pulse frequency measurement points (vertical dotted lines) are marked. (**B**) Arterial resistance index (ARI) was measured at baseline (pre-diet) and after 1 month of HFD feeding (post-diet) in external and internal carotids by Doppler ultrasonography in IrP and P3 groups (N=4/group). (**C**) Representative thin-layer chromatography of external and internal carotids and bar graphs showing the cholesteryl ester (CE)/free cholesterol (FC) ratio in carotids. Results are shown as mean ± SD. ***P<0.001, P3 *versus* control. *P<0.005 versus pred-diet; #P<0.005 versus IrP-rabbits.

B-mode ultrasound images of rabbit carotid arteries did not show thickening or calcification indicative of the presence of atherosclerotic lesions. Our available technology was not suitable for performing high-resolution studies (40-50 MHz) capable of detecting early atherosclerotic lesions in carotids. Doppler measurements were used to obtain the arterial resistance index (ARI), an indirect hemodynamic parameter that indicates the resistance to blood flow in a vascular bed distal to the points of measurement: the systolic (1, 3, and 5), diastolic (2, 4, and 6), and pulse frequency measurement points (vertical dotted lines) (marked in Figure 10A). ARI was measured at baseline (pre-diet) and after one month of feeding rabbits HFD (post-diet) into the external and internal carotids from the IrP and P3-immunized rabbits. There were no differences in terms of the basal measurements between the groups. HFD increased ARI in both the external and internal carotid arteries of the IrP rabbits. However, in the P3-immunized rabbits, HFD only slightly induced ARI in the internal carotid artery (Figure 10B). As in the aorta, HFD dramatically induced cholesteryl ester accumulation in the carotids of IrP rabbits and anti-P3 Abs significantly reduced CE accumulation in the external but not in the internal carotid artery (Figure 10C).

## Discussion

**Fig 11:**
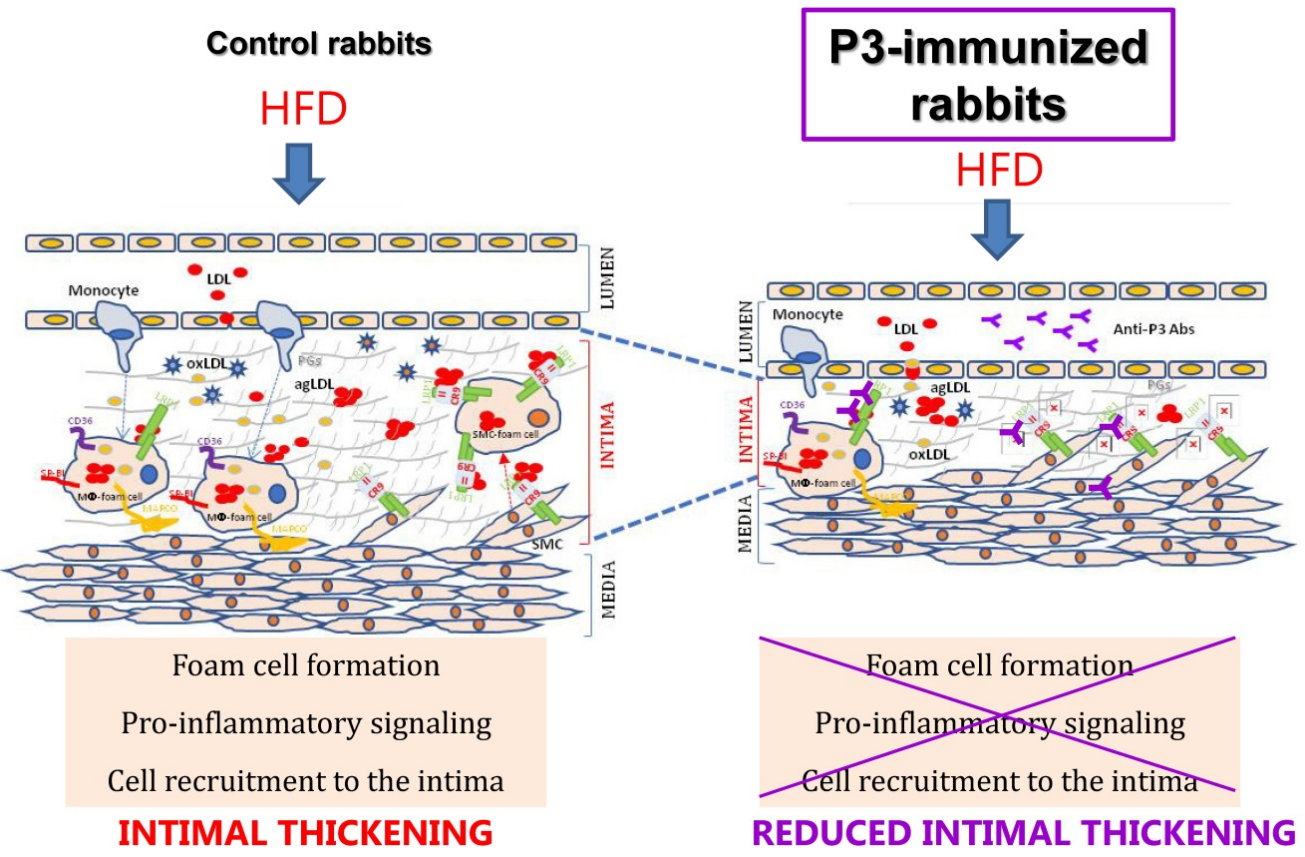
Graphical Abstract

In this study, we revealed the significant reduction of two surrogate markers of atherosclerosis, aortic ^18^F-FDG cellular metabolism and carotid resistance index, upon the P3 immunization of rabbits. Immunohistochemical, confocal, and molecular studies showed that P3 immunization efficiently counteracted the formation of fatty streaks due to the high efficacy of Anti-P3 Abs in preventing foam cell formation and their coupled pro-inflammatory signaling involved in monocyte recruitment and VSMC migration (summarized in the graphical abstract, Fig. 11).

Previous studies have consistently shown that LRP1 plays a crucial role in the maintenance of the vascular function and in atheroprotection due to its participation in signaling pathways that limit the vascular smooth muscle cell proliferative activity 12,13, apoptosis/efferocytosis and the pro-inflammatory signal of macrophages 14-16.The essential LRP1's role in signaling prompted us to develop antibodies with capacity to specifically block the binding of aggregated LDL. In the present study, we show that Anti-P3 Abs normalized aortic LRP1 levels until the levels found in the aorta of chow fed rabbits. Therefore, this strategy to modulate LRP1 function should not alter the binding of essential LRP1 ligands and coactivators.

HFD is known to promote atherosclerosis. Here, we found that, in agreement with previous studies 32,36, one month of HFD strongly promoted hypercholesterolemia and led to early atherosclerotic lesions or fatty streaks in NZW rabbits. In our rabbit experimental model, HFD increased circulating CE levels to aproximately 55 mM (2115 mg/dL), mainly transported by VLDL and LDL in the rabbit serum. Considering that the percentage of the protein in lipoproteins is similar to humans, LDL and VLDL in HFD-fed rabbits are extremely enriched in cholesteryl esters compared to human LDLs. In fact, the exposure of hMΦ and hcVSMC to HFD serum (0.5%) for only 2 hours caused a tremendous intracellular CE accumulation, in agreement with the high CE accumulation that we observed in the aorta of this rabbit model. In line with our results in rabbits, an increased atherogenicity of large CE-enriched ApoB-100 lipoproteins has been previously reported in monkeys and pigs 37,38. Remarkably, large LDLs have been associated with increased coronary artery disease in humans 39,40. The excessive cholesteryl ester load of these lipoproteins *per se* seems not to be a primary factor determining their increased atherogenicity. Core cholesteryl ester and triglyceride seem to cause serious alterations in ApoB-100 conformation, a key determinant of the LDL affinity by the LDLR 41,42. Here, the high capacity of rabbit CE-enriched ApoB-100 lipoproteins to cause intracellular CE accumulation in human macrophages and VSMC suggest that they are not taken up through the LDLR, a receptor downregulated by intracellular CE levels. Unexpectedly, anti-P3 Abs, designed to inhibit agLDL, not only counteracted CE accumulation in the rabbit vasculature, where LDL aggregation could have been facilitated by proteoglycans of the arterial intima, but also in hMΦ and hcVSMC that have been directly exposed to VLDL and LDL of the rabbit serum. The high efficacy of Anti-P3 Abs to block serum-induced hMΦ and hcVSMC cholesteryl ester loading suggest that rabbit large VLDL and/or LDL (CE-enriched ApoB-100 lipoproteins) could be interacting with the human LRP1, probably through the same domain than aggregated LDL. Futher molecular studies are indeed required to know how CE-enriched large VLDLs and/or LDLs influence ApoB-100 conformation and whether the epitope of ApoB-100 generated in these lipoproteins share structural similarities with that generated in aggregated LDL. An important challenge for future research will be to design molecular tools suitable to reduce the vascular impact of the majority of atherogenic lipoproteins.

Differently to aorta, where HFD only upregulates vascular cholesteryl ester content, HFD increased both hepatic cholesteryl esters and triglycerides. The differential impact of HFD serum in the neutral lipid content of aorta and liver could be associated to the particular lipoproteins that mainly interact with vascular and hepatic tissues. In vascular cells (macrophages and VSMC), LRP1 supposedly interacts greatly with agLDL 4-6. In hepatic cells, LRP1 mainly interacts with ApoE-enriched lipoproteins such as chylomicrons 43. It is known that ApoB-100 and ApoE interact with LRP1 receptor through different clusters. The new epitope generated in ApoB-100 during LDL aggregation interacts with cluster II (CR9 domain) 24 while ApoE interacts with cluster III (domain CR17) 44,45. The different lipoproteins that reach aortas and livers may explain why P3 immunization reduces cholesteryl ester accumulation in the vasculature (aorta and carotids) but not in liver. The CR9 and CR17 domains are so spatially distant that it is structurally impossible for anti-P3 antibodies to cause any effect on the binding of ApoE-enriched lipoproteins. This could explain why P3 immunization did not exert any effect on the HFD-induced neutral lipid accumulation in the liver. The liver is a key organ that modulates whole-body lipid and lipoprotein metabolism and consequently circulating lipid and lipoprotein profile 46. Therefore, the lack of impact of the Anti-P3 Abs in the hepatic neutral lipid content could be a determinant of the similar lipoprotein profile between groups.

Molecular, immunohistochemical and confocal microscopy experiments showed that Anti-P3 Abs decreased HFD-induced TNFR1 overexpression in vasculature and cells. Macrophage-foam cells have been reported to secrete extracellular vesicles that determine the proliferative and migratory capacity of SMC 47. Moreover, TNFR1 promotes monocyte recruitment and SMC proliferation in the pro-atherosclerotic arterial wall 23. Therefore, the reduced TNFR1 levels that we observed in Anti-P3 treated macrophages and VSMCs could indeed contribute to limit the recruitment of monocytes and VSMCs into the arterial intima.

The impact of HFD on NF-kB (p65) phosphorylation reported in this study is in line with previous results showing that an excessive input of nutrition activates NF-kB signaling in macrophages 48. Similar HFD effects were observed in the phosphorylation of NF-kB (p65) phosphorylation in VSMC. The high efficiency of Anti-P3 Abs to counteract HFD-induced NF-kB (p65) phosphorylation in hMΦ and hcVSMC, suggest a high potential of these Abs to modulate atherosclerosis. Abbate's group has elegantly shown that, by counteracting NF-kB pro-inflammatory signaling, LRP1 activation with SP16 agonist leads to a cardioprotective signal that reduces infarct size 20,21. SP16 has been designed on the basis of the motif present in protease-inhibitor complexes, which have been reported not to be recognized by the consecutive CR8/CR9 modules of cluster II 25. The specific interaction of anti-P3 Abs with CR9 domains ensures high specificity of effects, and importantly, guarantees zero interference with the agonists of the LRP1 anti-inflammatory signaling. Of note that anti-P3 Abs did not exert any effect on these basal LRP1 levels, suggesting that Anti-P3 Abs reduced HFD-induced pro-inflammatory signaling without disturbing the atheroprotective LRP1 signaling in the vasculature.

Taken together, our results highlight the strategic therapeutic value of Anti-P3 Antibodies to inhibit diet-induced cellular cholesteryl ester loading and coupled inflammation in the vasculature.

In line with previous studies showing that ^18^F-FDG uptake reaches maximal values during early foam cell formation 35, in the current study, we also showed that the anti-atherosclerotic efficiency of P3 immunization on early lesions can be tracked by PET/ CT. Complementary confocal microscopy analysis showed that reduced ^18^F-FDG uptake in the aorta of P3-immunized rabbits occurred concomitantly with a reduced presence of macrophages and SMCs in the arterial intima. Although cellular metabolic activity in PET/CT has been assumed to be mainly caused by the high number of macrophages in atherosclerotic plaques 28, more recent studies have demonstrated that SMCs also play a crucial role in plaque PET/CT activity due to their high capacity to take up glucose when exposed to pro-inflammatory cytokines 49.

While imaging for the detection of atherosclerotic lesions at different stages has been extensively used for rabbit aortas, less investigated has been the impact of HFD on the development of atherosclerosis in carotids of this animal model, and especially through imaging techniques. Here, we showed that one month of HFD increased the resistance index (RI) of both external and internal carotids despite the lack of direct evidence of atherosclerotic plaques in carotids. In carotid arteries, a significant correlation between RI and the degree of generalized atherosclerosis has been previously shown in humans 50. Here, we demonstrated the efficacy of P3-immunization in reducing carotid RI, although the effect was different between the external and internal carotids (higher for the external carotids). In fact, further carotid lipidic analysis showed that P3 immunization reduced cholesteryl ester accumulation on the external but not on the internal carotid. Differential anatomy and hemodynamics could likely explain the differential impact of P3 immunization on these carotids. On the basis of aortic PET/CT and carotid Doppler ultrasonography, our results revealed that P3 immunization efficiently reduces HFD-induced atherosclerosis in rabbits, at least in the early stages. Moreover, these results suggested that PET/CT and ultrasonography are imaging techniques that can track the effect of potential therapeutic P3 immunizations in other translational *in vivo* models and in humans.

### Limitations of the study

The main limitations of this study were that the efficacy of P3 immunization was not analyzed in the endothelium despite the crucial role of endothelial *lrp1* in atherosclerosis, glucose sensitivity and lipid profiles 51. Recently, it has been reported the feasibility of a surface-enhanced Raman scattering-antibody-functionalized gold nanoprobes (SERS-BFNP) molecular imaging platform for non-invasive detection of adhesion molecules in the endothelium of atherosclerotic lesions in *in vivo* models 52. In addition, the efficacy of P3 immunization was only tested in early atherosclerotic lesions despite the high relevance of the interaction of VSMC with LDL for the vulnerability of the atherosclerotic plaques 53. Finally, the technology used in the present study to detect the presence of atherosclerotic plaques in carotids has certain limitations.

## Conclusions

In conclusion, our study demonstrates that, by specifically blocking LRP1-mediated intracellular CE accumulation in vascular cells, Anti-P3 Abs counteract cellular pro-inflammatory signals that allow for the recruitment of monocytes and VSMC into the arterial intima of a rabbit model of HFD-induced atherosclerosis. Moreover, anti-P3 Abs are extremely effective to stop foam cell formation from human macrophages and human coronary vascular smooth muscle cells and the efficacy of P3 immunization can be tracked using non-invasive imaging techniques such as PET/CT and Doppler ultrasonography. These both aspects confer an enormous translational potential to Anti-P3 Abs.

## Supplementary Material

Supplementary figures.

## Abbreviations

AgLDL: aggregated LDL
ANOVA: analysis of variance
ApoA-I: apolipoprotein A-I
apoB-100: apolipoprotein B-100
ApoJ: apolipoprotein J
ARI: arterial resistance index
BSA: bovine serum albumin
CE: cholesteryl esters
CVD: cardiovascular disease
ELISA: enzyme-linked immunosorbent assay
FDG: 2-fluoro-2-deoxy-D-glucose
HFD: high-fat diet
IMT: intima-media thickness
KLH: Keyhole limpet haemocyanin
LDL: low-density lipoprotein
LRP1: low-density lipoprotein receptor-related protein 1
NF-kB: nuclear factor kB
NZW: New Zealand White
PET/CT: positron emission tomography/computed tomography
RI: resistance index
rSMC: rabbit smooth muscle cells
SMC: smooth muscle cells
SUV: standard uptake value
TNFR1: tumor necrosis factor receptor 1
