# Supplementary material for: Immunization with the Gly1127-Cys1140 amino acid sequence of the LRP1 receptor reduces atherosclerosis in rabbits. Molecular, immunohistochemical and nuclear imaging studies
Source: Theranostics. 2020 Feb 10;10(7):3263–80. doi: 10.7150/thno.37305 (PMC7053206; doi:10.7150/thno.37305)
Supplement: Supplementary file 1 — Supplementary figures. [file thnov10p3263s1.pdf]

Figure S1

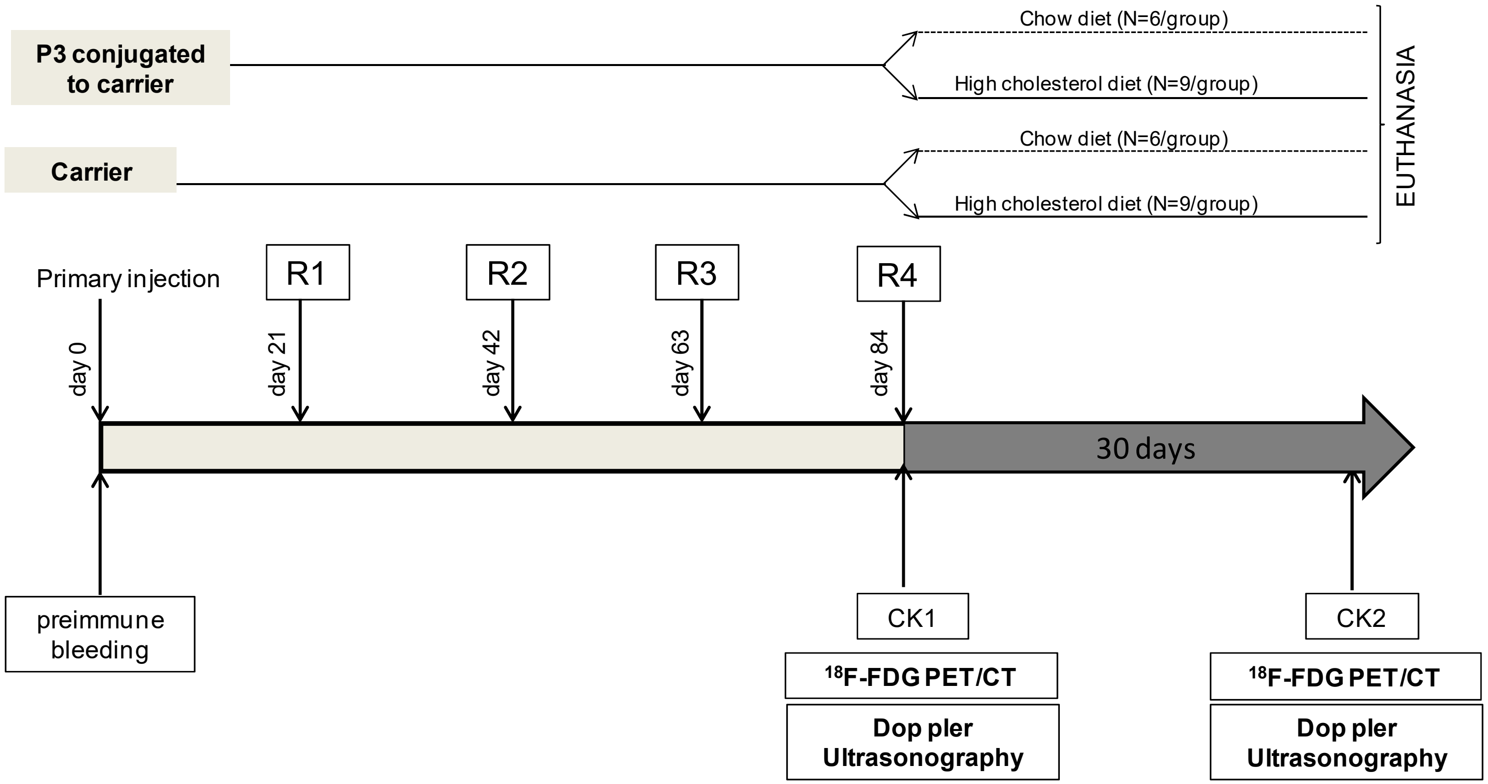

Figure S1. Study Design. Animals were acclimated for one week before the first immunization and immunized with a primary injection and four reminder doses (R1-R4) of IrP (irrelevant peptide) (IrP group; N=12) or P3 (P3 group; N=15) conjugated to the carrier every 21 days. An additional group of rabbits was injected with carrier alone (control group; N=3). At day 84, 18F-FDG PET/CT image scans, Doppler ultrasonography and circulating anti-P3 specific antibody determinations (CK1) were performed and considered as a reference of basal levels. Starting at the R4 time point, IrP and P3-immunized rabbits were randomly divided into normal diet group and high-fat diet (HFD)-fed group. Twelve rabbits (N=6 Irp-injected and N=6 P3-injected) continued fed on the chow diet, whereas fifteen rabbits (N=6 IrP-injected and N=9 P3-injected) and one rabbit control group (N=3 injected with carrier alone) received HFD for 30 days. After one month, final imaging PET/CT scans, Doppler measurements and circulating anti-P3 specific antibody determinations (CK2) were performed. At day 114, animals were then euthanized, and tissues (aorta, carotids and liver) were processed for immunohistochemistry, confocal microscopy and molecular studies. CK1: check point 1 (pre-diet); CK2: check point 2 (post-diet

Figure S2

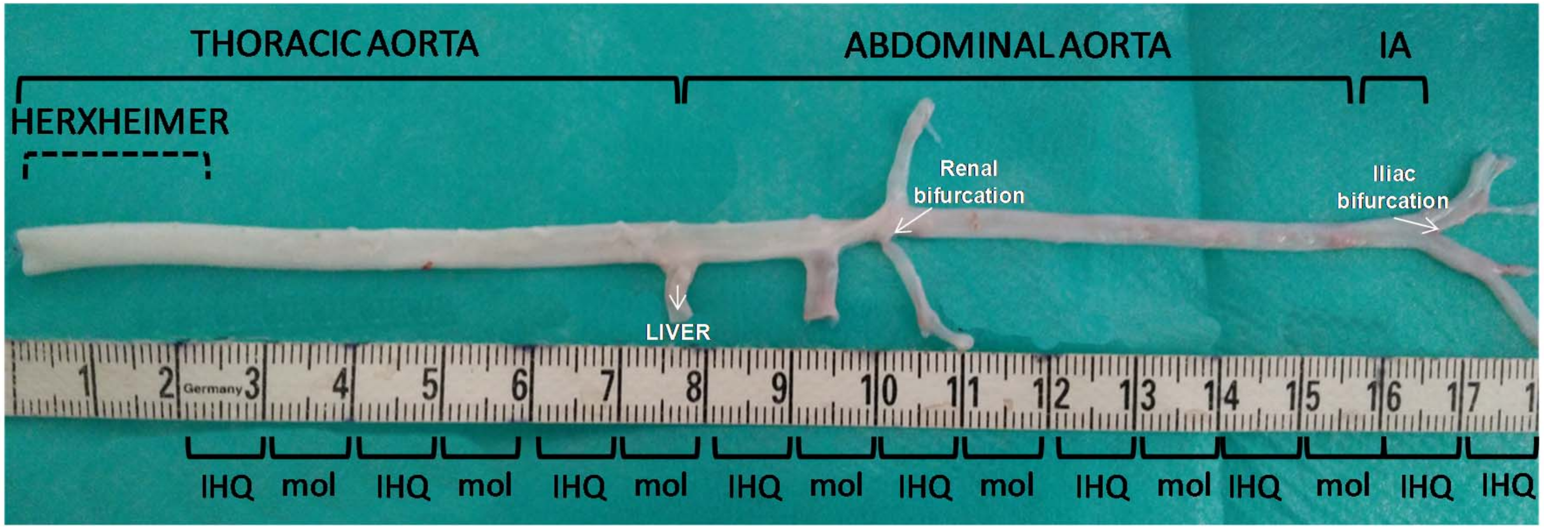

Figure S2. Representative image of isolated aorta indicating the consecutive sections (1 cm) used for immunohistochemistry (IHQ) and molecular studies (mol). IA: Iliac bifurcation.

Figure S3

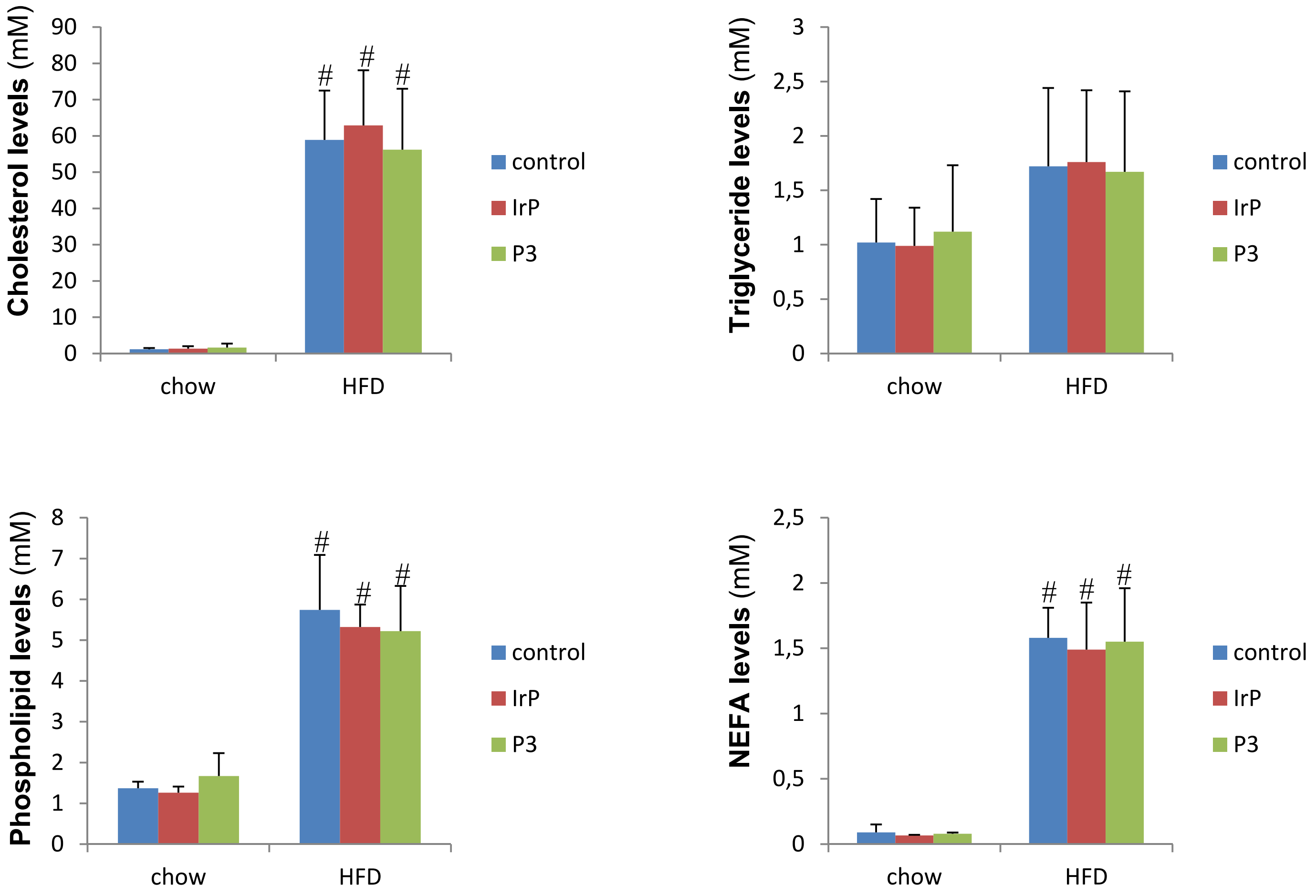

Figure S3. Circulating lipid levels in control and P3-immunized rabbits. Bar graphs showing cholesterol (A), triglycerides (B), non-esterified fatty acids (NEFA) (C) and phospholipid (D) levels in serum from control, IrP and P3-immunized rabbits. # P<0.005 versus chow diet.

Figure S4

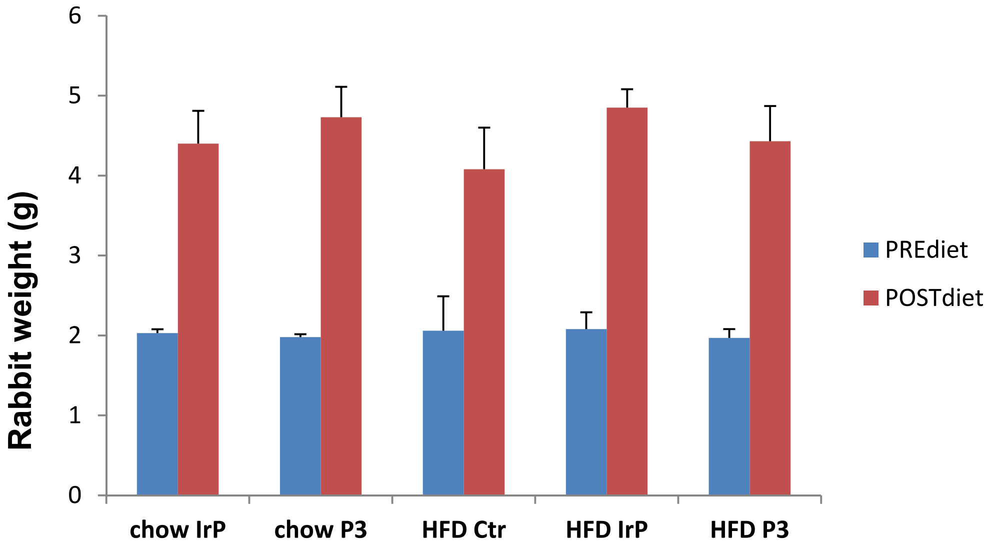

Figure S4. Rabbit weight gain in pre-and post-diet time points in the different studied groups

Figure S5

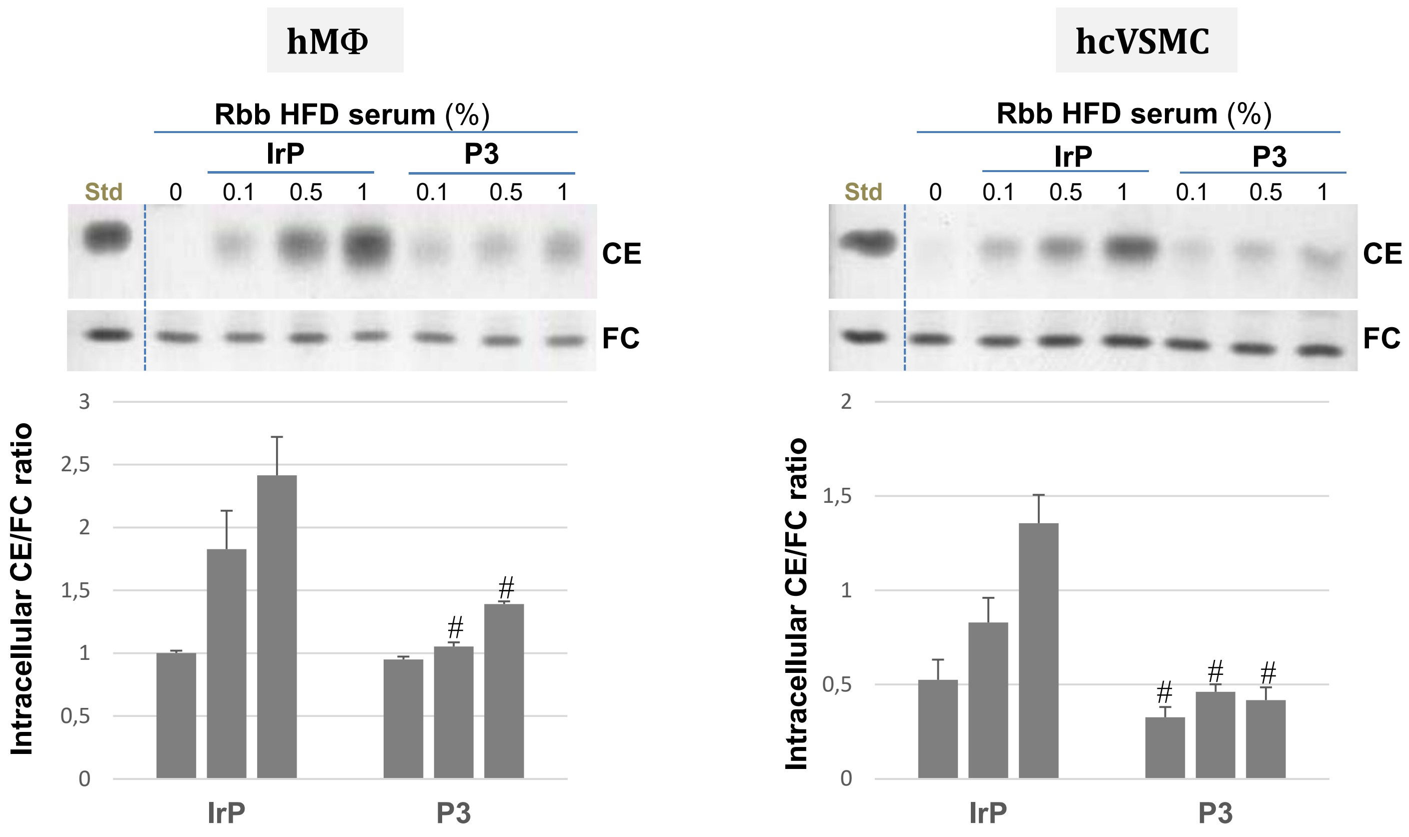

Figure S5. Effect of HFD serum from IrP and P3-immunized rabbits in the intracellular CE/FC ratio of human macrophages (hMΦ) and human coronary vascular smooth muscle cells (hcVSMC). Quiescent hMΦ and hcVSMC were exposed for increasing serum dose (0.1%, 0.5% and 1%) for two hours. Cells were then exhaustively washed and collected in NaoH 0.1N for lipid extraction followed by TLC. Bar graphs show the cholesteryl ester (CE) /free cholesterol ratio. Data are shown as mean ± SEM of three experiments performed in duplicate. # P<0.005 versus IrP.

Figure S6

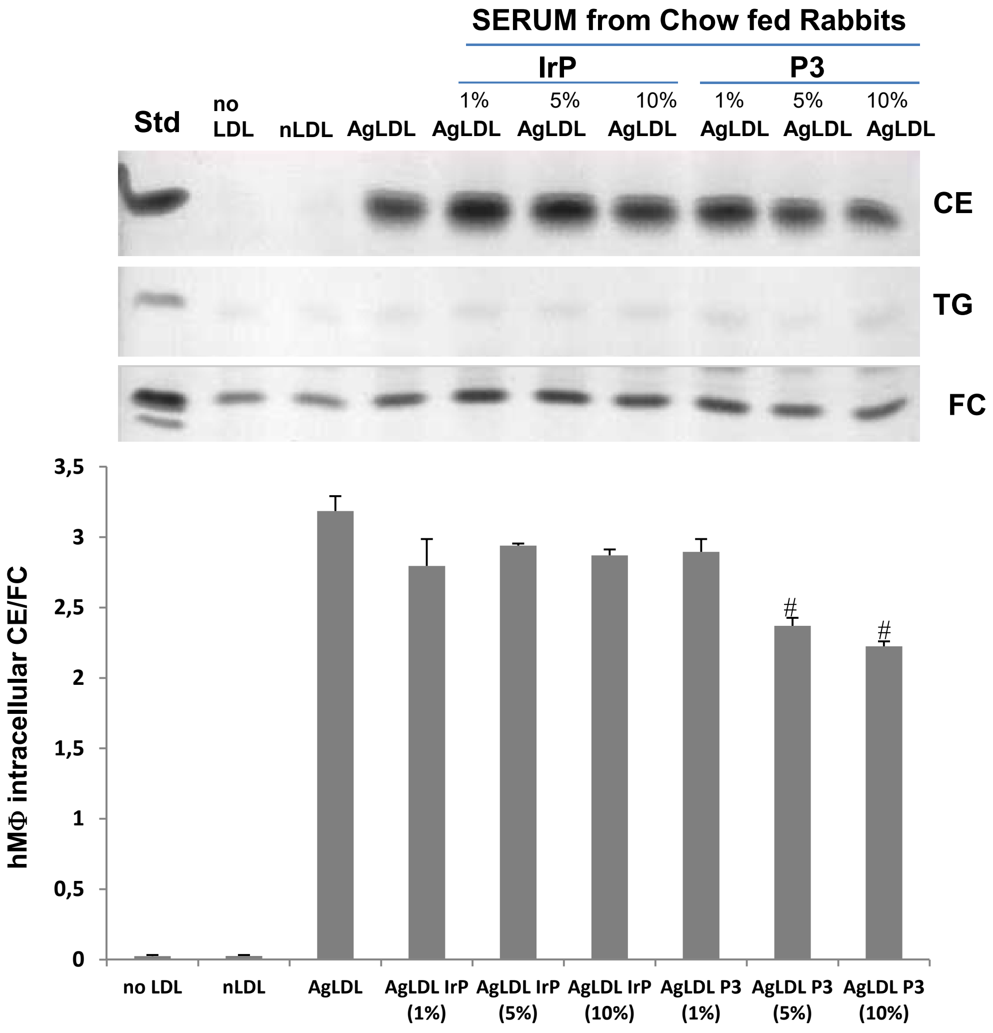

Figure S6. Effect of chow serum from IrP and P3-immunized rabbits in the intracellular CE/FC ratio induced by aggregated LDL in human macrophages (hMΦ). Quiescent hMΦ were exposed to aggregated LDL (100 µg/mL, 2 hours) in the presence of increasing dose of chow serum from IrP or P3 rabbits (1%, 5% and 1%). Cells were then exhaustively washed and collected in NaoH 0.1N for lipid extraction followed by TLC. Bar graphs show the cholesteryl ester (CE) /free cholesterol ratio. Data are shown as mean ± SEM of three experiments performed in duplicate. # P<0.005 versus IrP.

Figure S7

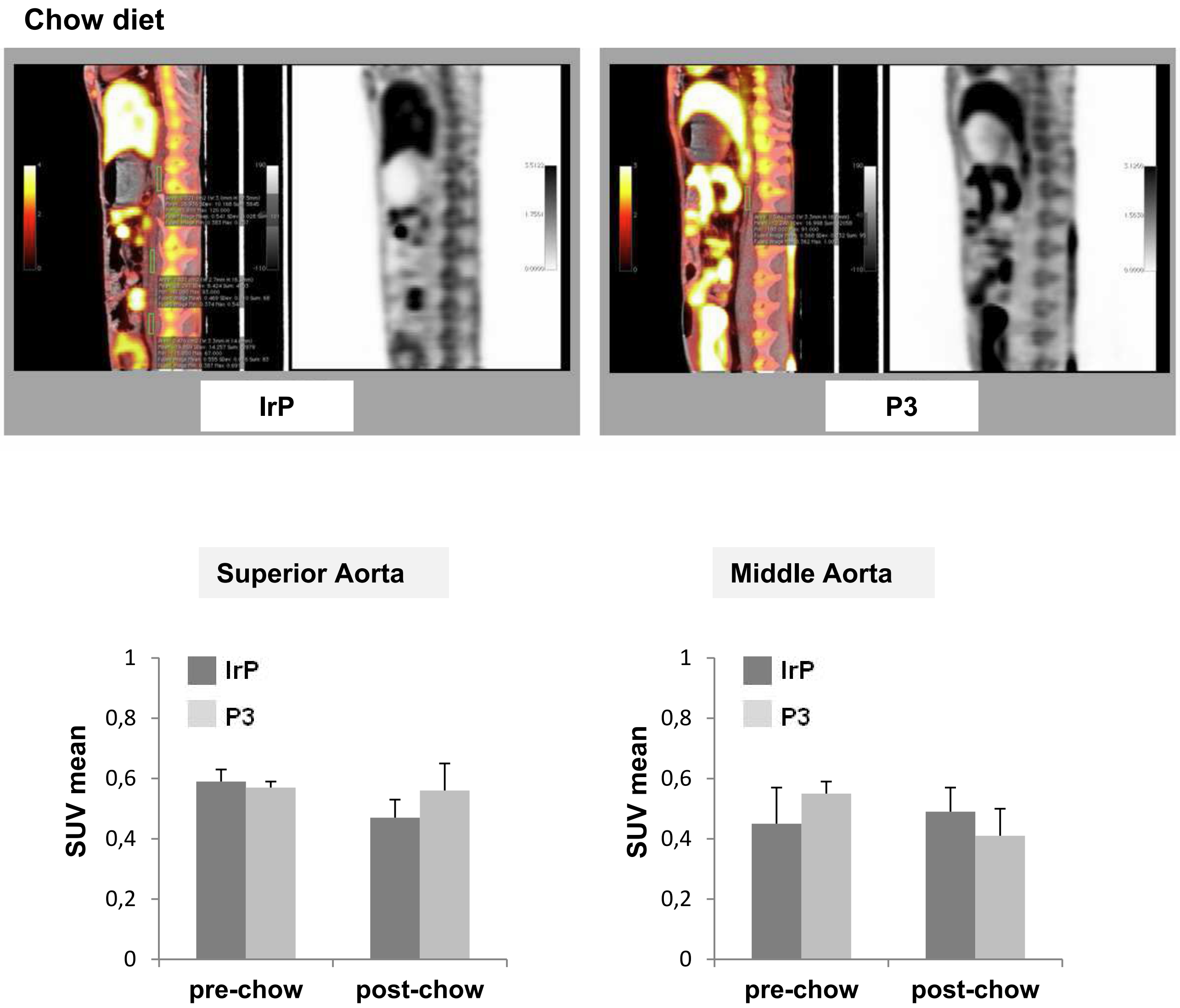

Figure 9. P3 immunization did not exert any effect on basal aortic 18F-FDG uptake. (A) Representative PET/CT longitudinal images of the aorta in control and P3 immunized rabbits fed chow diet. (B) Graphs showing the SUVmean values at pre-diet and post-diet time points in the upper and middle parts of the aorta in chow-fed rabbits (IrP and P3 groups) (N=3/group)..
